# Supplementary material for: CytA, a reductase in the cytorhodin biosynthesis pathway, inactivates anthracycline drugs in Streptomyces
Source: Commun Biol. 2019 Dec 6;2:454. doi: 10.1038/s42003-019-0699-5 (PMC6897945; doi:10.1038/s42003-019-0699-5)
Supplement: Supplementary file 1 — Supplementary Information [file 42003_2019_699_MOESM1_ESM.pdf]

Supplementary Figures

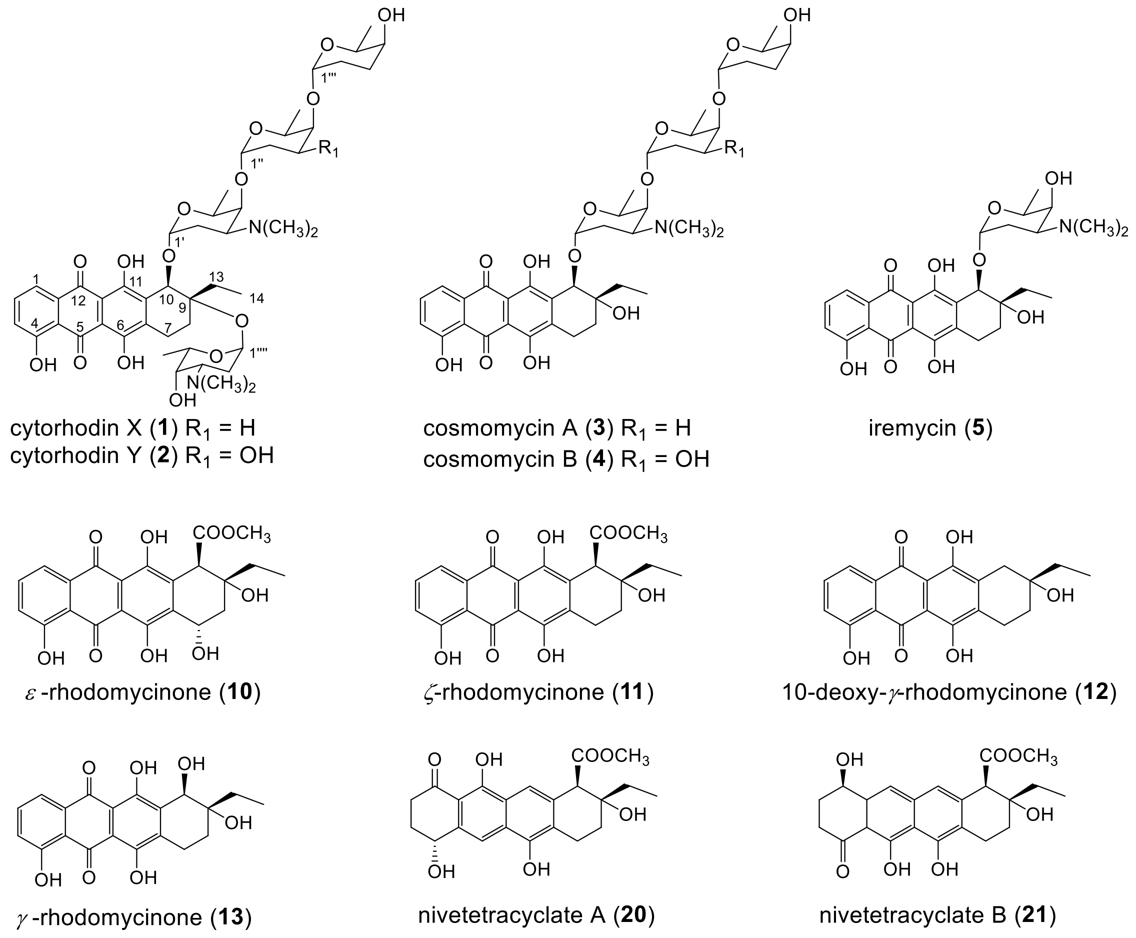

Supplementary Figure 1. Chemical structures of natural C-7 saturated containing anthracyclines and their aglycones.

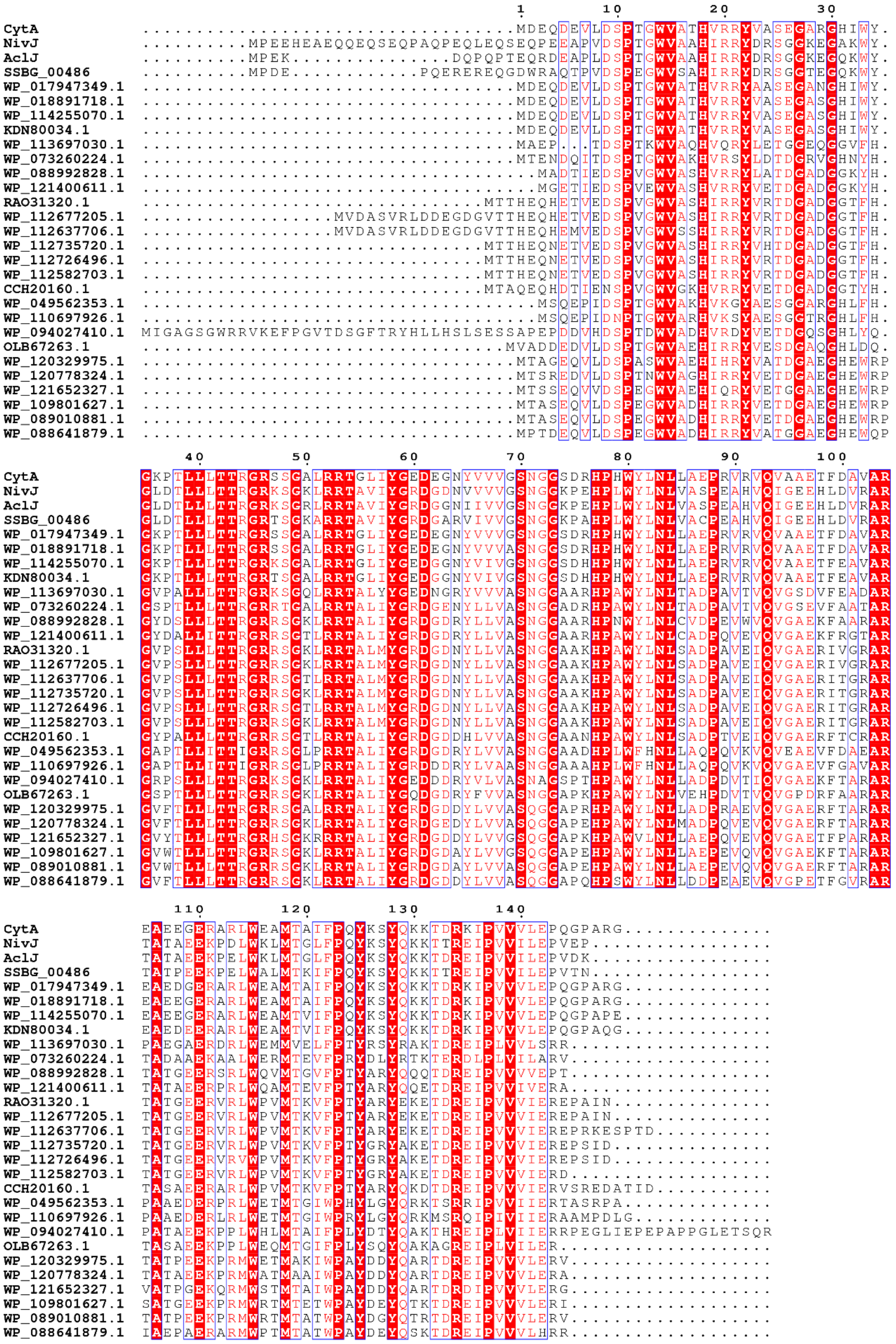

Supplementary Figure 2. Multiple sequence alignments for CytA with its homologues (NivJ, AclJ, SSBG_00486, WP_017947349.1, WP_018891718.1, WP_114255070.1, KDN80034.1, WP_113697030.1, WP_094027410.1, WP_073260224.1, WP_088992828.1, WP_121400611.1, RAO31320.1, WP_112677205.1, WP_112637706.1, WP_112735720.1, WP_112726496.1, WP_112582703.1, CCH20160.1, WP_049562353.1, WP_110697926.1, WP_094027410.1, OLB67263.1, WP_120329975.1, WP_120778324.1, WP_121652327.1, WP_109801627.1, WP_089010881.1, WP_088641879.1)

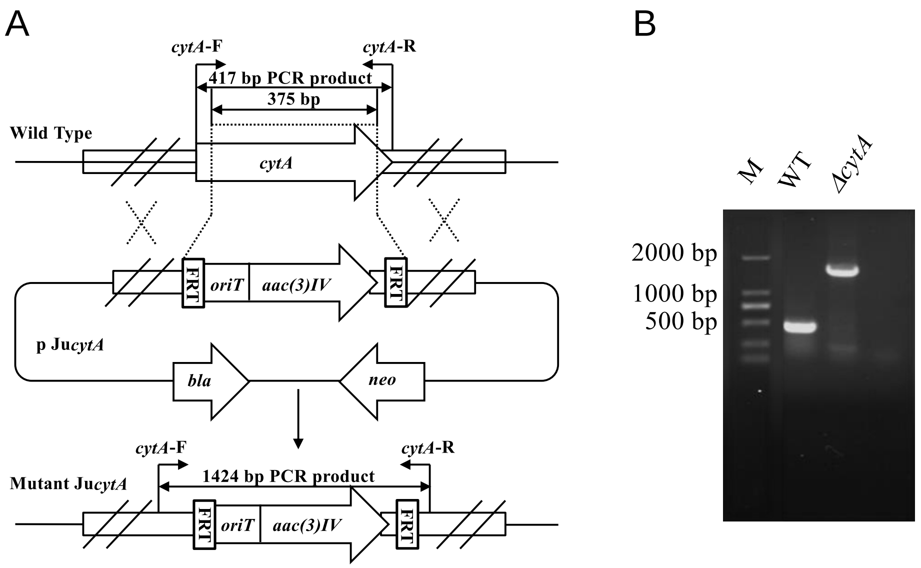

Supplementary Figure 3. Construction and gel electrophoresis analyses of mutant strain Δ*cytA*. (A) Construction of mutant Δ*cytA* and predicted PCR fragment size from wild-type and mutant; (B) Verification of the Δ*cytA* mutant by PCR. M: DNA molecular ladder, DNA marker DL2000; WT: using the genomic DNA of *Streptomyces* sp. SCSIO 1666 as template; Δ*cytA*: using the genomic DNA of *cytA* mutant as template.

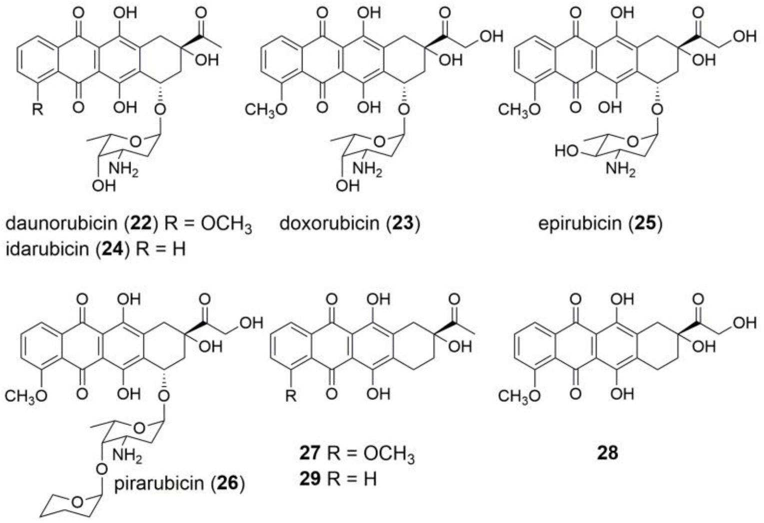

Supplementary Figure 4. Chemical structures of clinically employed anthracycline antitumor drugs and their 7-deoxyanthracyclines.

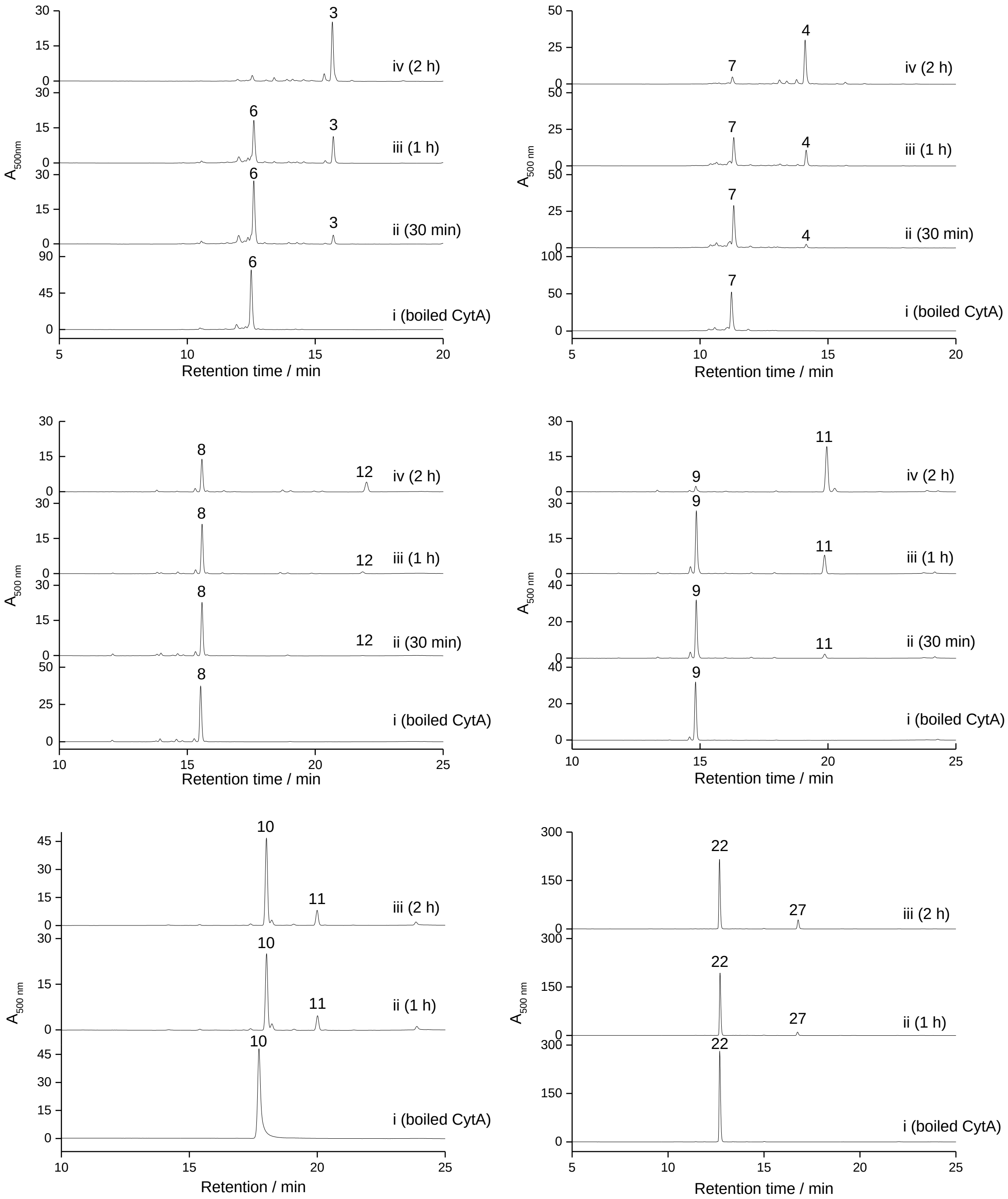

3
iv (2 h)
6
3
iii (1 h)
6
3
ii (30 min)
6
i (boiled CytA)
A_{500nm}
Retention time / min
4
7
iv (2 h)
7
4
iii (1 h)
7
4
ii (30 min)
7
i (boiled CytA)
$A_{500 nm}$
Retention time / min
8
12
iv (2 h)
8
12
iii (1 h)
8
12
ii (30 min)
8
i (boiled CytA)
$A_{500 nm}$
Retention time / min
11
9
iv (2 h)
9
11
iii (1 h)
9
11
ii (30 min)
9
i (boiled CytA)
$A_{500 nm}$
Retention time / min
10
11
iii (2 h)
10
11
ii (1 h)
10
i (boiled CytA)
$A_{500 nm}$
Retention / min
22
27
iii (2 h)
22
27
ii (1 h)
22
i (boiled CytA)
$A_{500 nm}$
Retention time / min

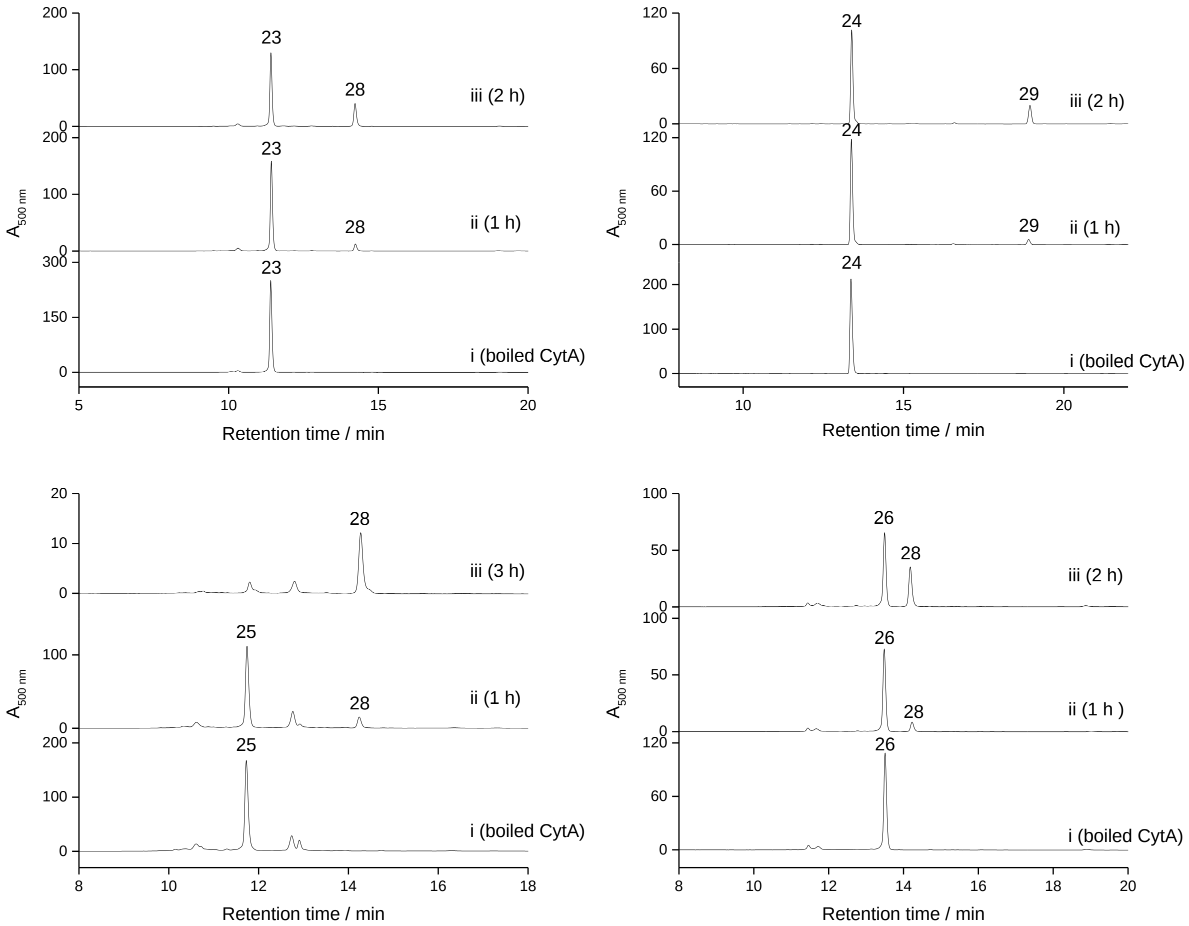

Supplementary Figure 5. Time course analyses for CytA-catalyzed modification of **6–10** and **22–26**.

A. HR-ESI-MS spectrum for compound daunorubicin (**22**) and its reductive cleavage product 7-deoxydaunomycinone (**27**).

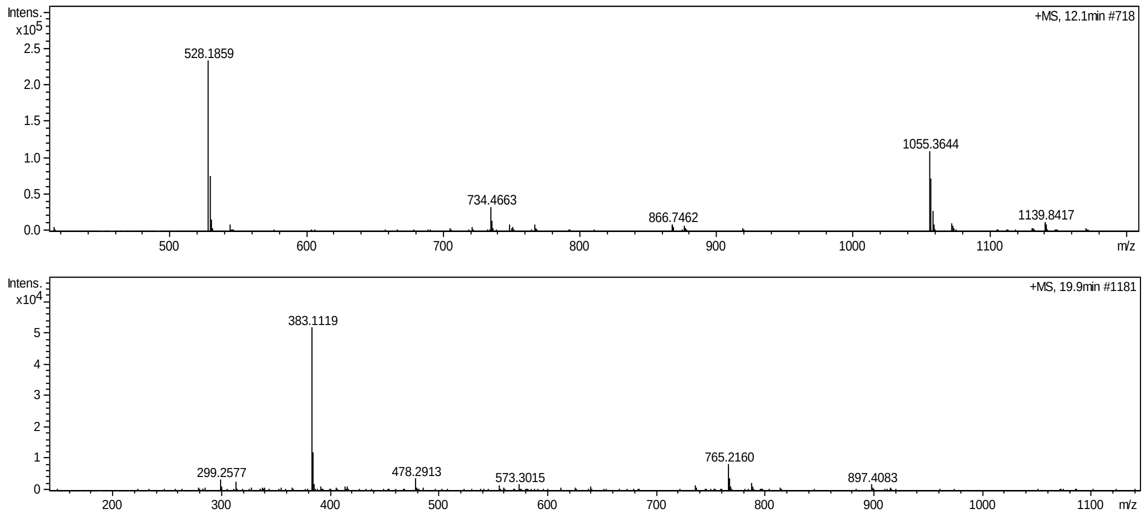

B. HR-ESI-MS spectrum for compound doxorubicin (**23**) and its reductive cleavage product 7-deoxydoxorubicinone (**28**).

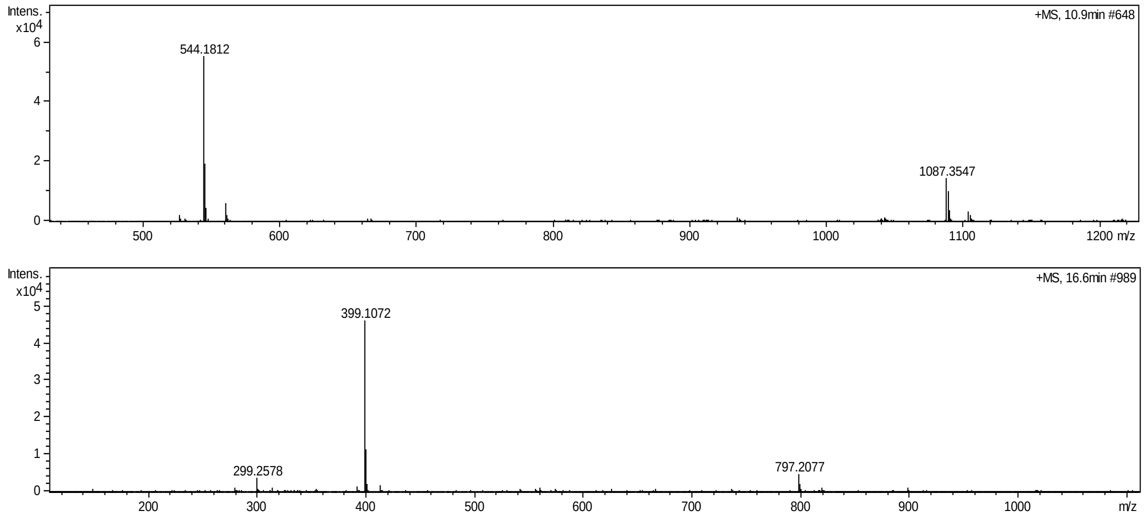

C. HR-ESI-MS spectrum for compound idarubicin (**24**) and its reductive cleavage product 7-deoxyidarubicinone (**29**).

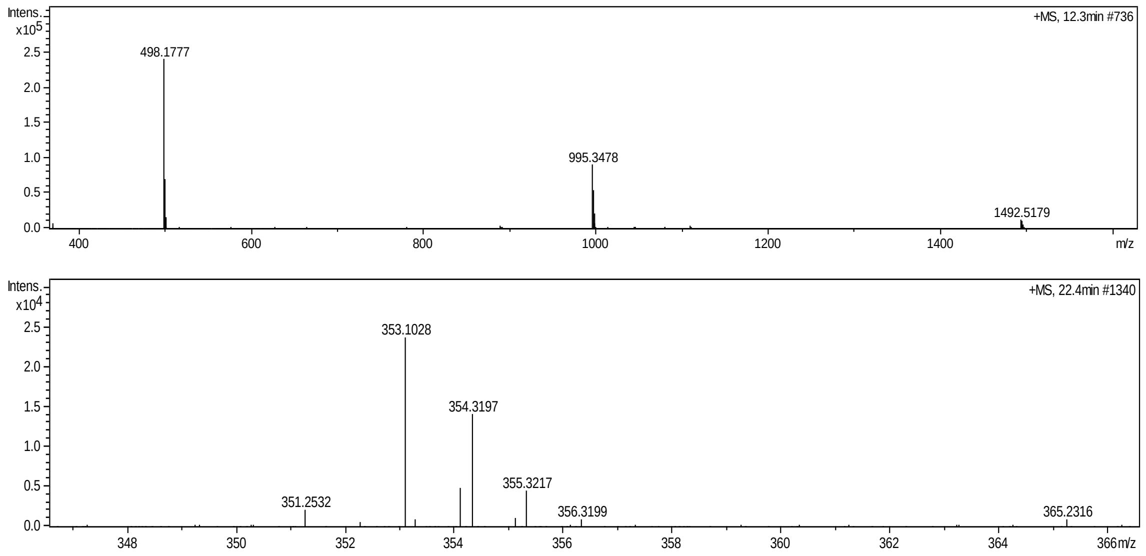

D. HR-ESI-MS spectrum for compound epirubicin (**25**) and its reductive cleavage product 7-deoxydoxorubicinone (**28**).

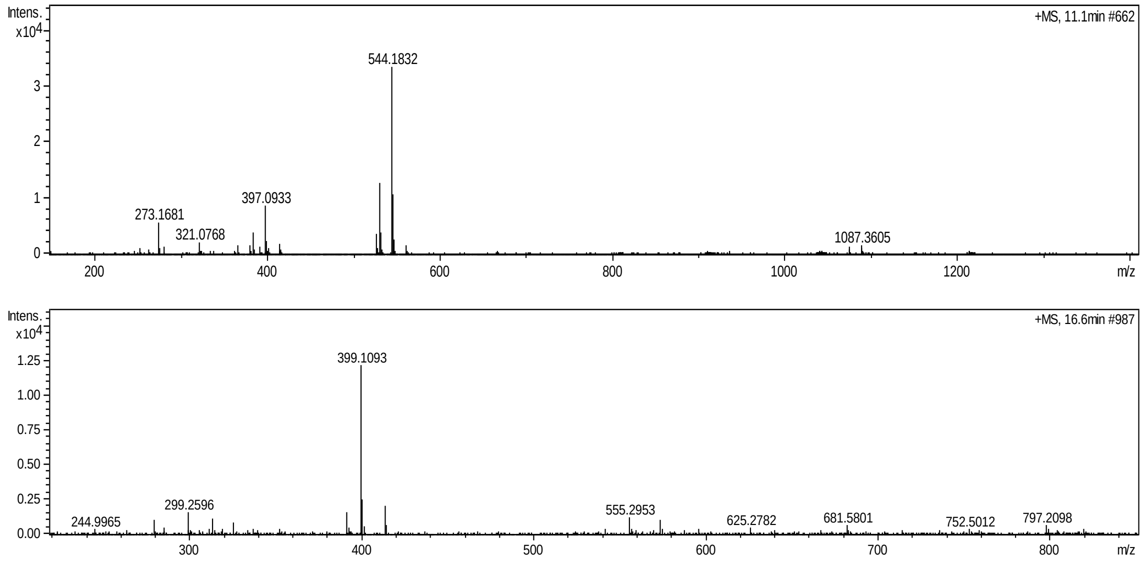

E. HR-ESI-MS spectrum for compound pirarubicin (**26**) and its reductive cleavage product 7-deoxydoxorubicinone (**28**).

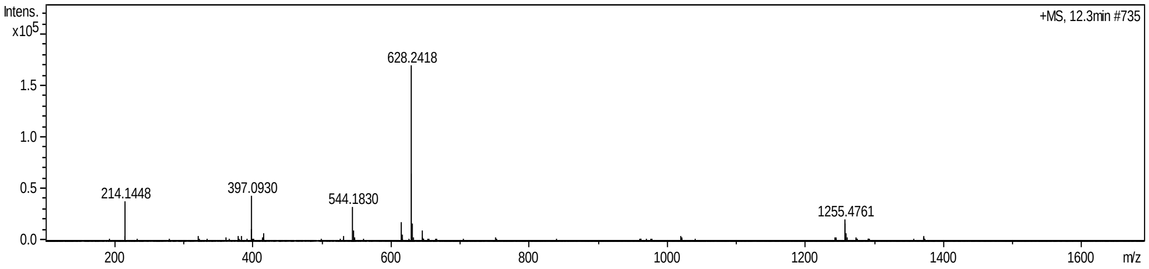

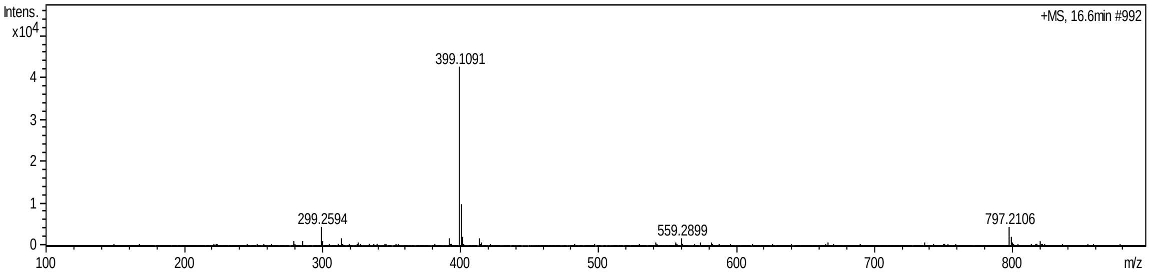

F. HR-ESI-MS spectrum for compound **8**

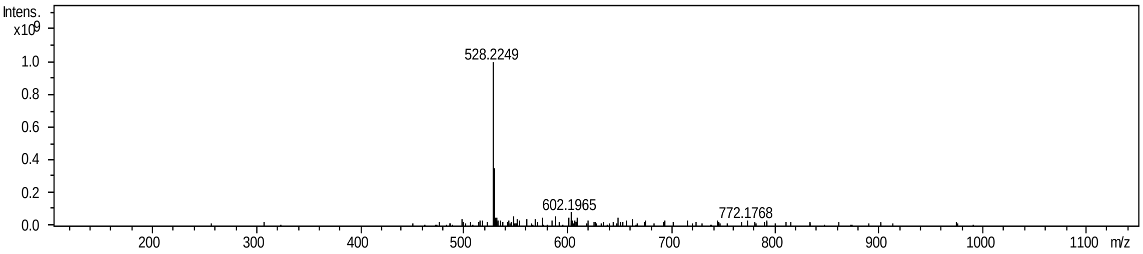

Supplementary Figure 6. The HR-ESI-MS spectra for the five clinically used anthracycline drugs and their reductive cleavage products, and compound **8**.

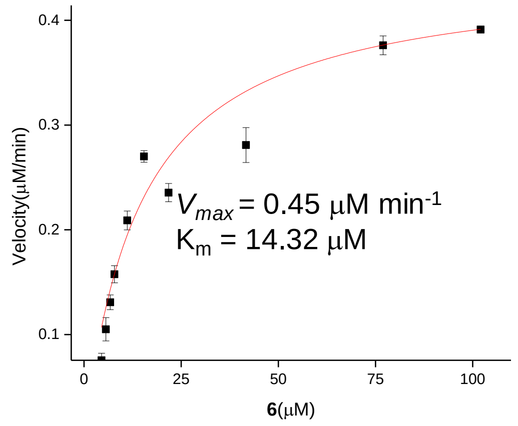
Velocity(μM/min)
0.1
0.2
0.3
0.4
0
25
50
75
100
6(μM)
V_{max} = 0.45 μM min^{-1}
K_m = 14.32 μM

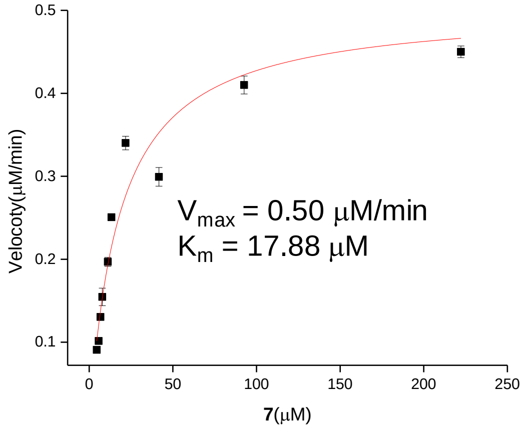
Velocoty(μM/min)
0.1
0.2
0.3
0.4
0.5
0
50
100
150
200
250
7(μM)
V_{max} = 0.50 μM/min
K_m = 17.88 μM

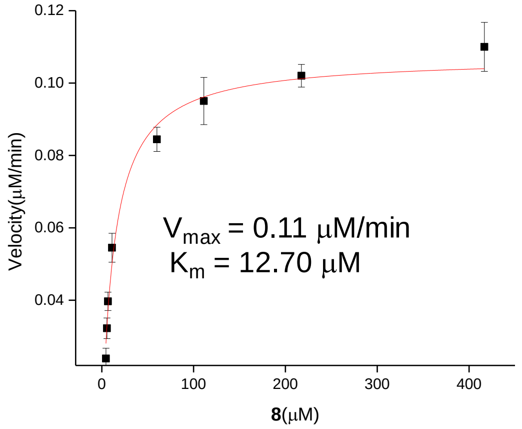
Velocity(μM/min)
0.04
0.06
0.08
0.10
0.12
0
100
200
300
400
8(μM)
V_{max} = 0.11 μM/min
K_m = 12.70 μM

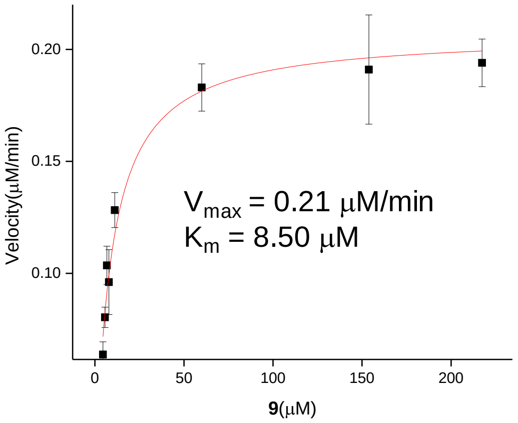
Velocity(μM/min)
0.10
0.15
0.20
0
50
100
150
200
9(μM)
V_{max} = 0.21 μM/min
K_m = 8.50 μM

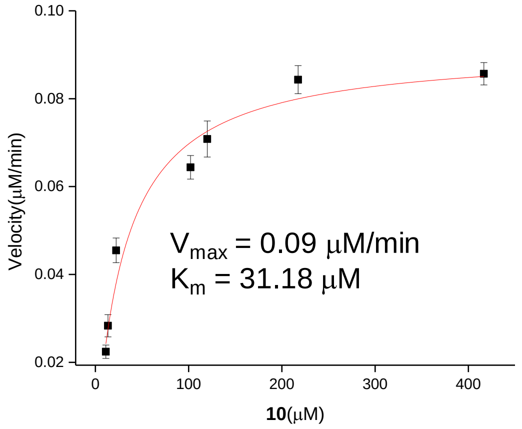
Velocity(μM/min)
0.02
0.04
0.06
0.08
0.10
0
100
200
300
400
10(μM)
V_{max} = 0.09 μM/min
K_m = 31.18 μM

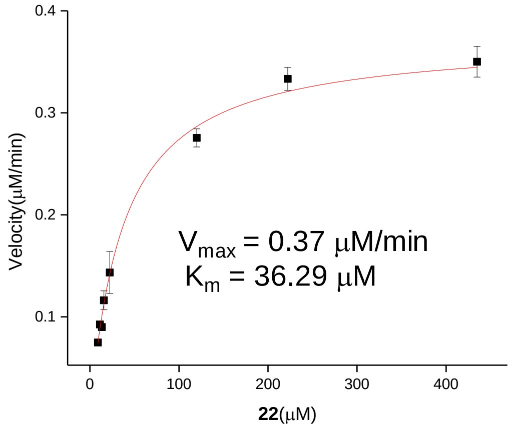
Velocoty(μM/min)
0.1
0.2
0.3
0.4
0
100
200
300
400
22(μM)
V_{max} = 0.37 μM/min
K_m = 36.29 μM

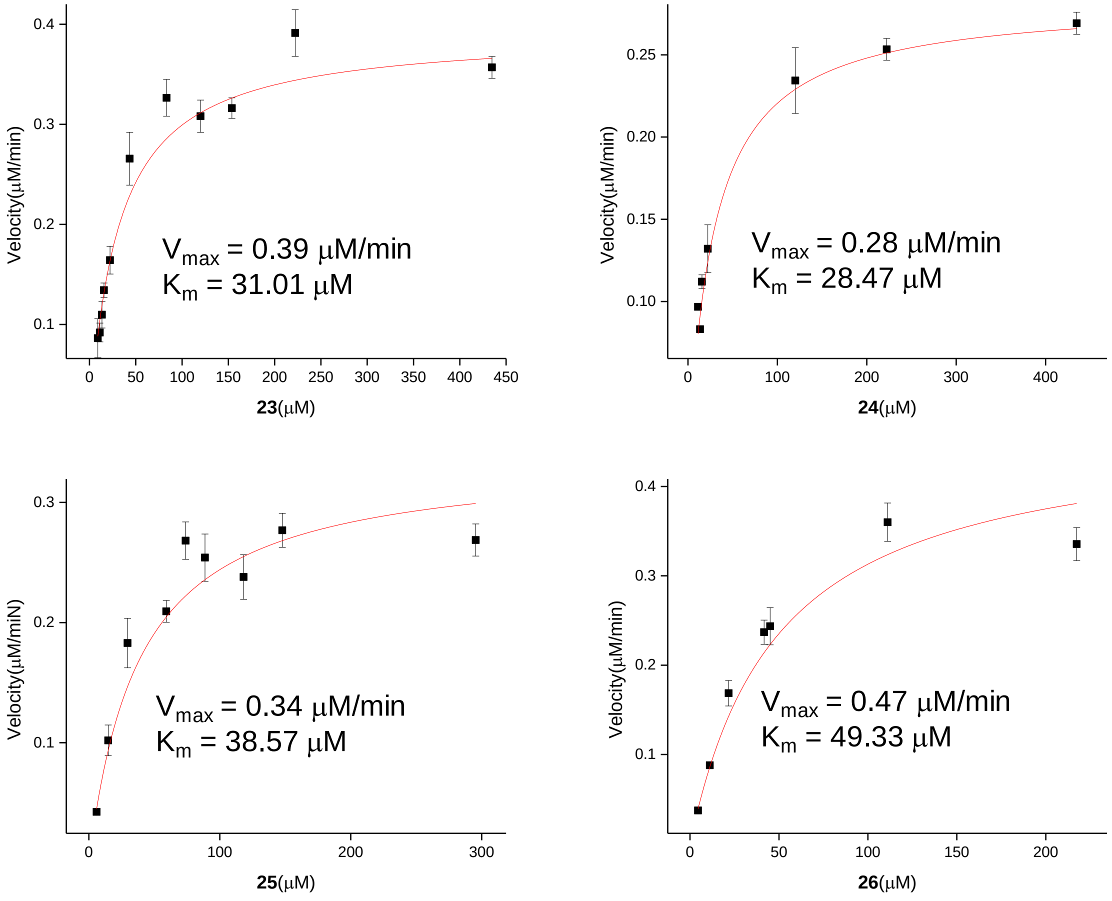

Supplementary Figure 7. Kinetic parameters for CytA processing of cosmomycin C (**6**), cosmomycin D (**7**), 10-decarbomethoxy-ε-rhodomycin (**8**), ε-rhodomycin (**9**), ε-rhodomycinone (**10**), daunorubicin (**22**), doxorubicin (**23**), idarubicin (**24**), epirubicin (**25**) and pirarubicin (**26**). Kinetics were performed in triplicate and each data point represents the mean of the three independent assays with error bars representing the standard deviation.

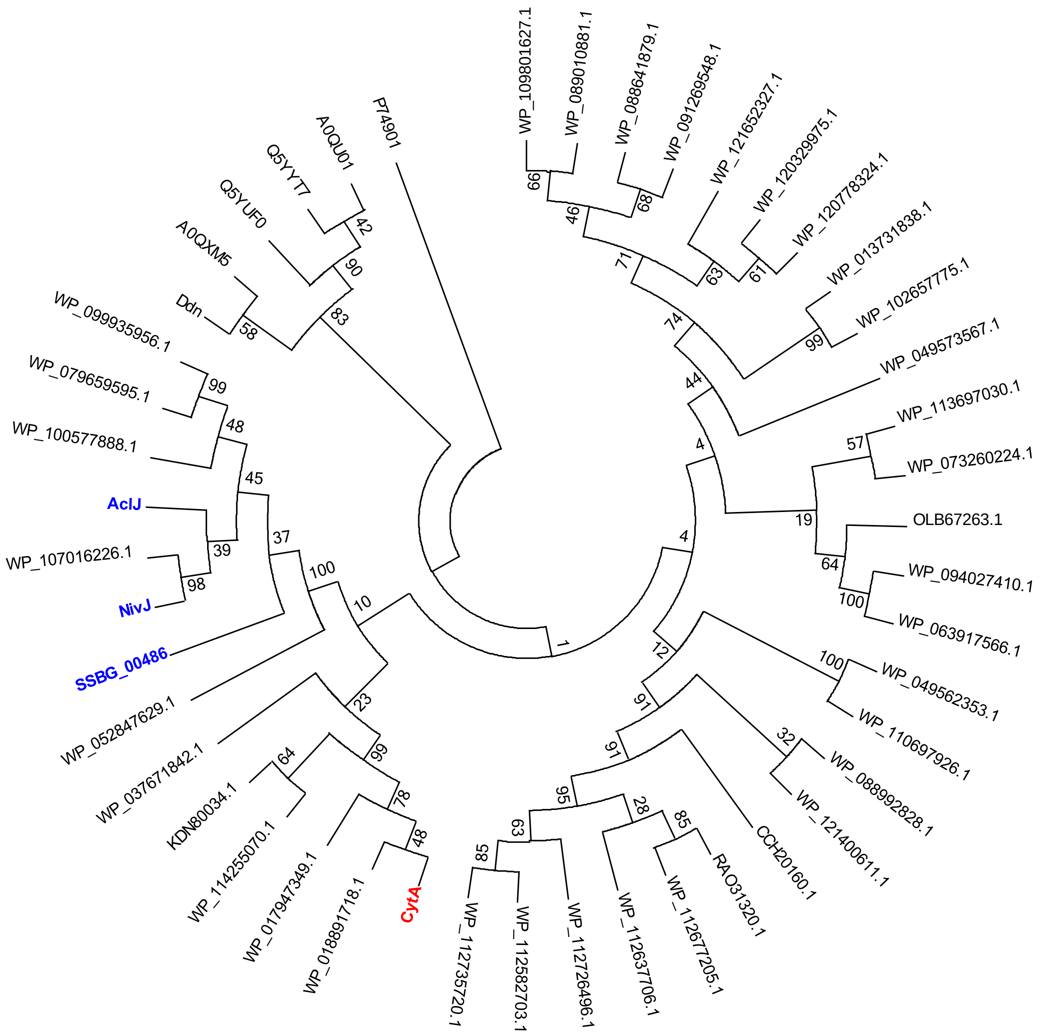

Supplementary Figure 8. Phylogenetic analysis of CytA, its homologs, and other selected members of the nitroreductase family (selected from the NR and Uniprot databases). The phylogenetic tree was generated by MEGA6 using the maximum likelihood method with a bootstrap test of 500 replicates. Numbers next to the branches represent the percentage of replicate trees in which the shown topology was reached.

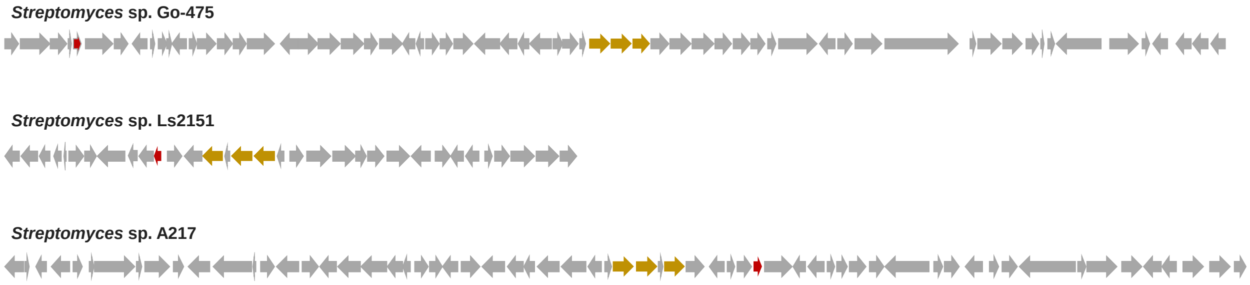

Supplementary Figure 9. Representative potentail type II PKSs for anthracycline-like biosynthetic gene clusters encoding for CytA homologues. Genes encoding CytA homologues are shown in red. Genes encoding type II PKSs homologues are shown in gold.

Supplementary Figure 10. ^1H NMR (700 MHz) spectrum of compound **6** in $CDCl_3$

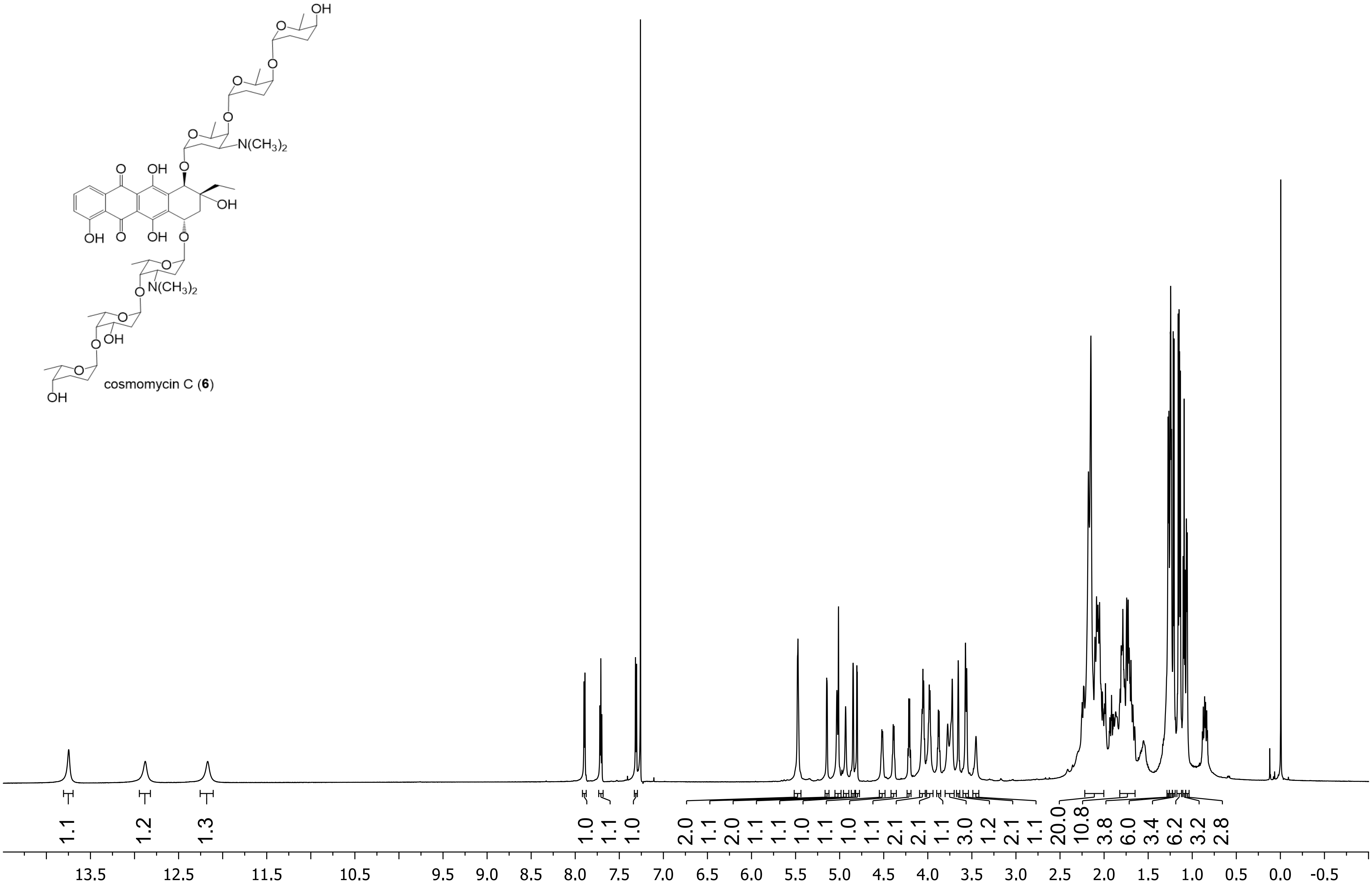

Supplementary Figure 11. ^{13}C NMR (175 MHz) spectrum of compound **6** in $CDCl_3$

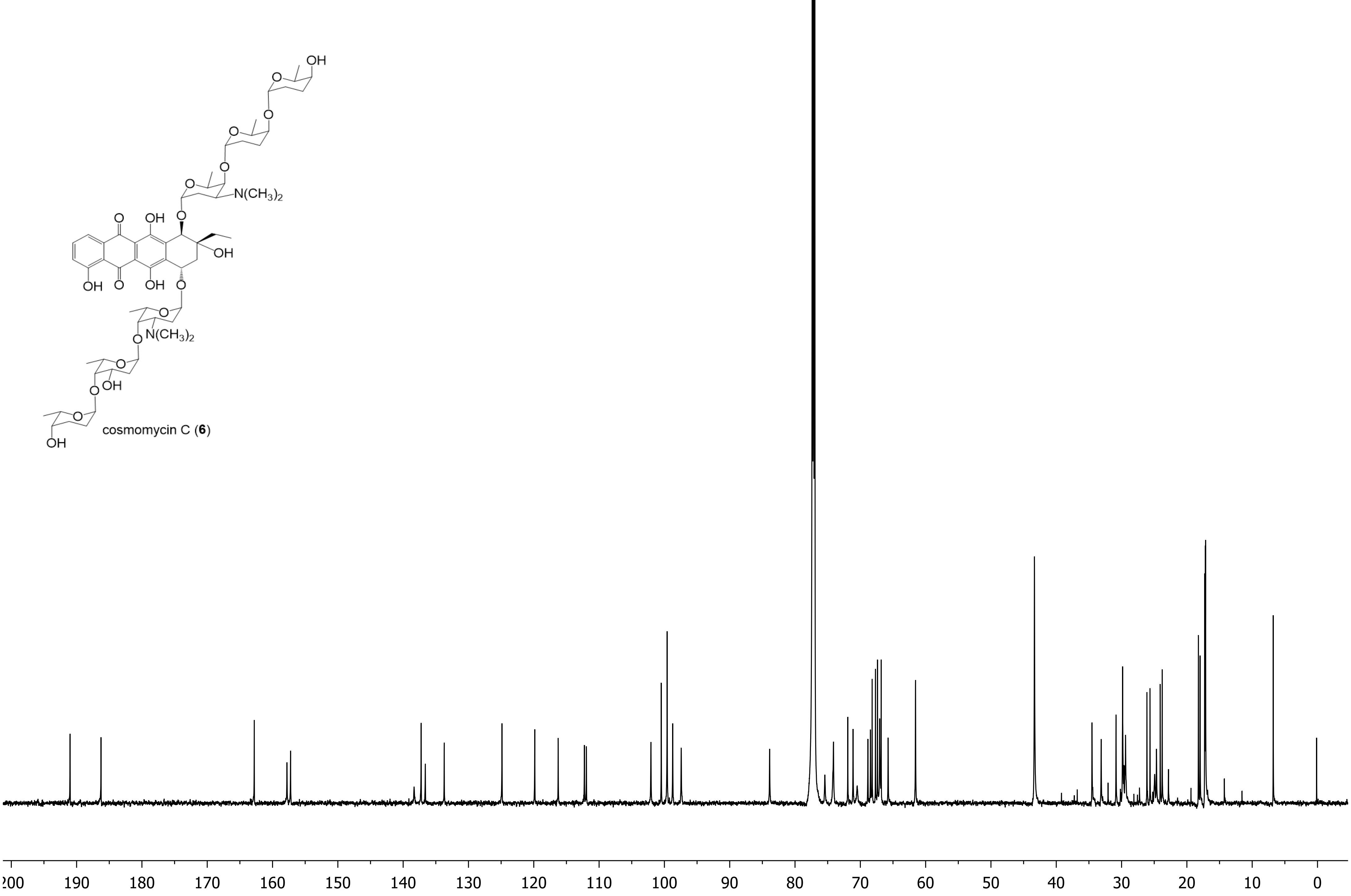

Supplementary Figure 12. DEPT 135 NMR (175 MHz) spectrum of compound **6** in $CDCl_3$

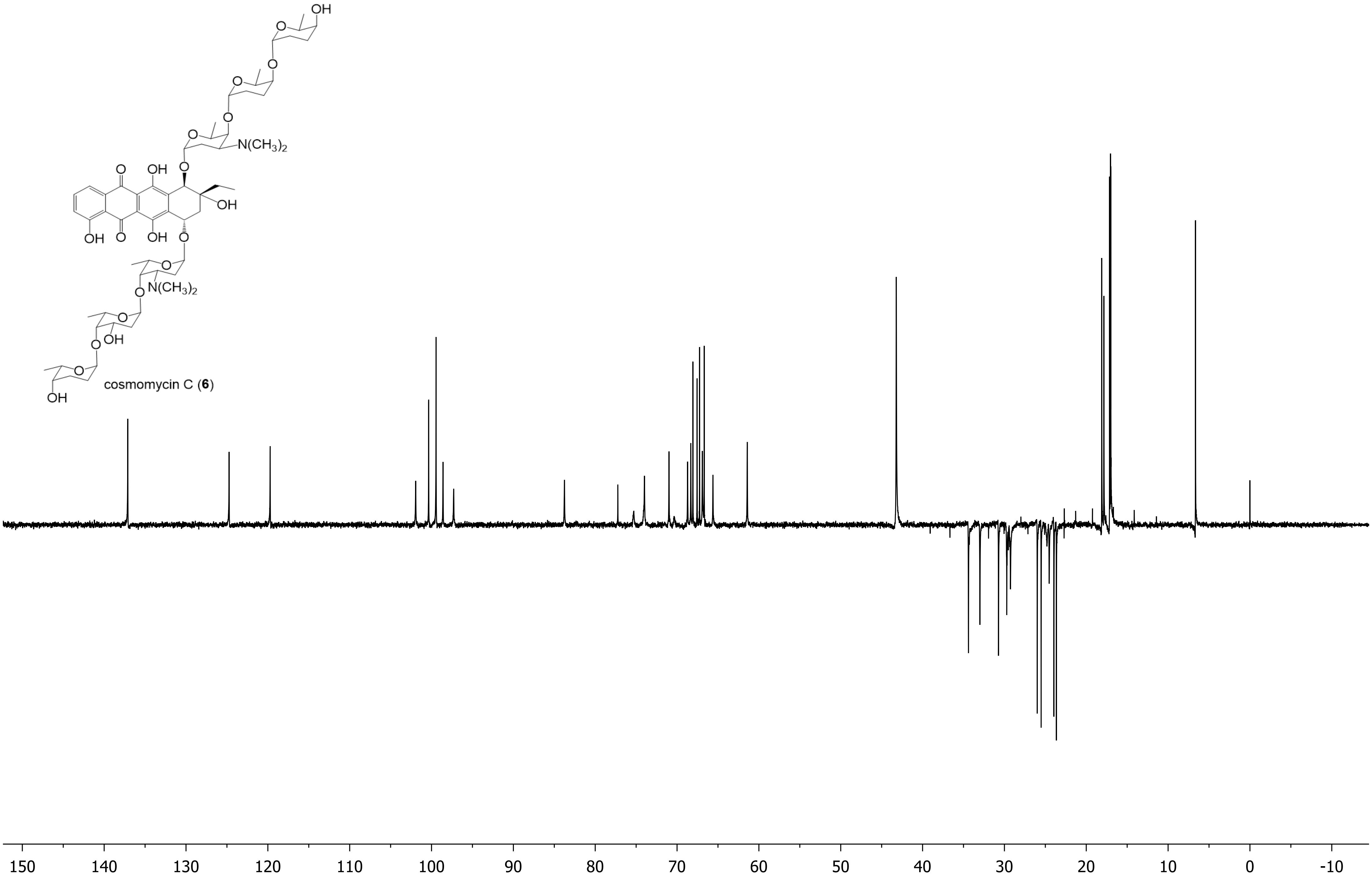

Supplementary Figure 13. ^{1}H-^{1}H COSY spectrum of compound **6** in $CDCl_3$

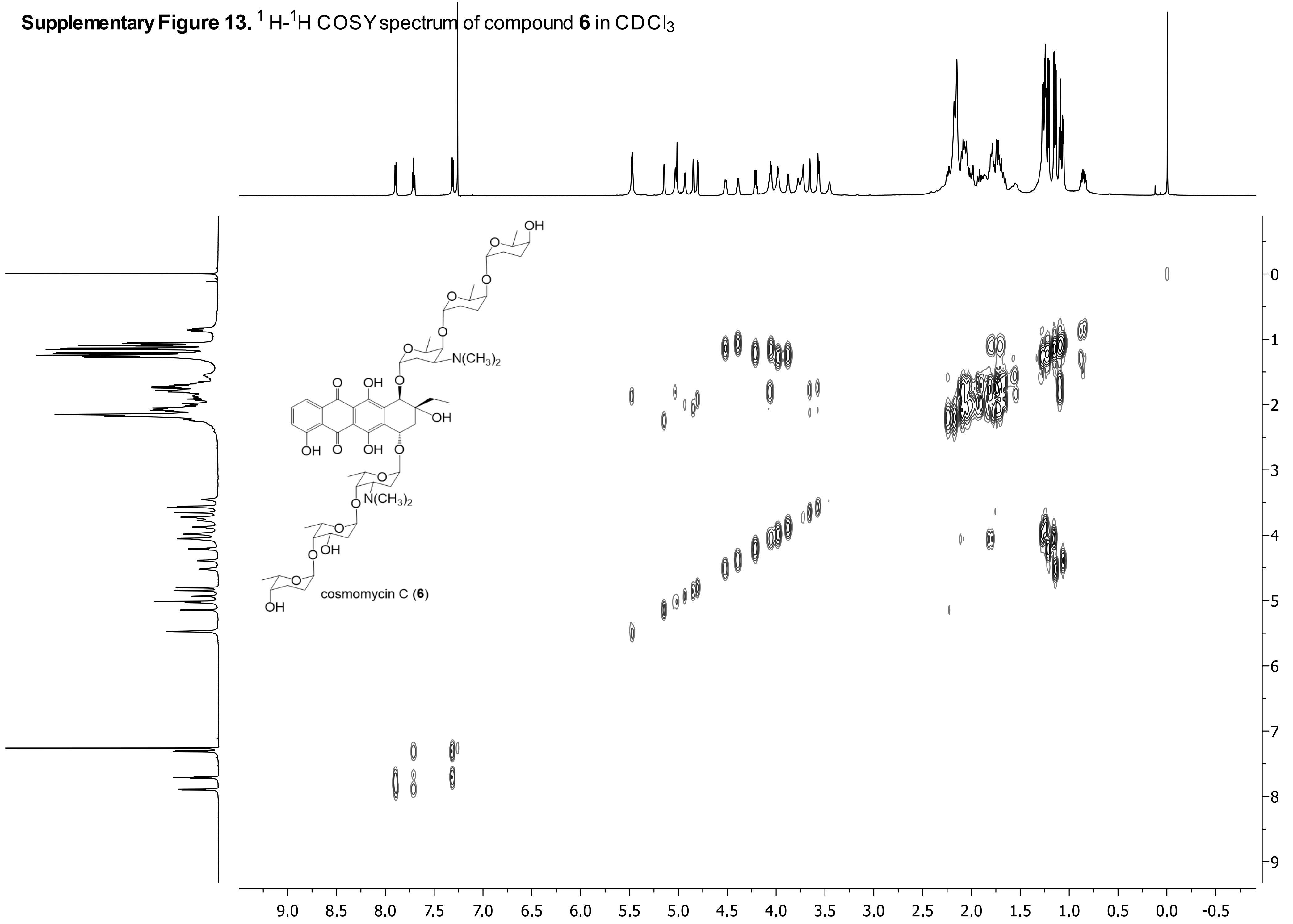

Supplementary Figure 14. HSQC spectrum of compound **6** in $CDCl_3$

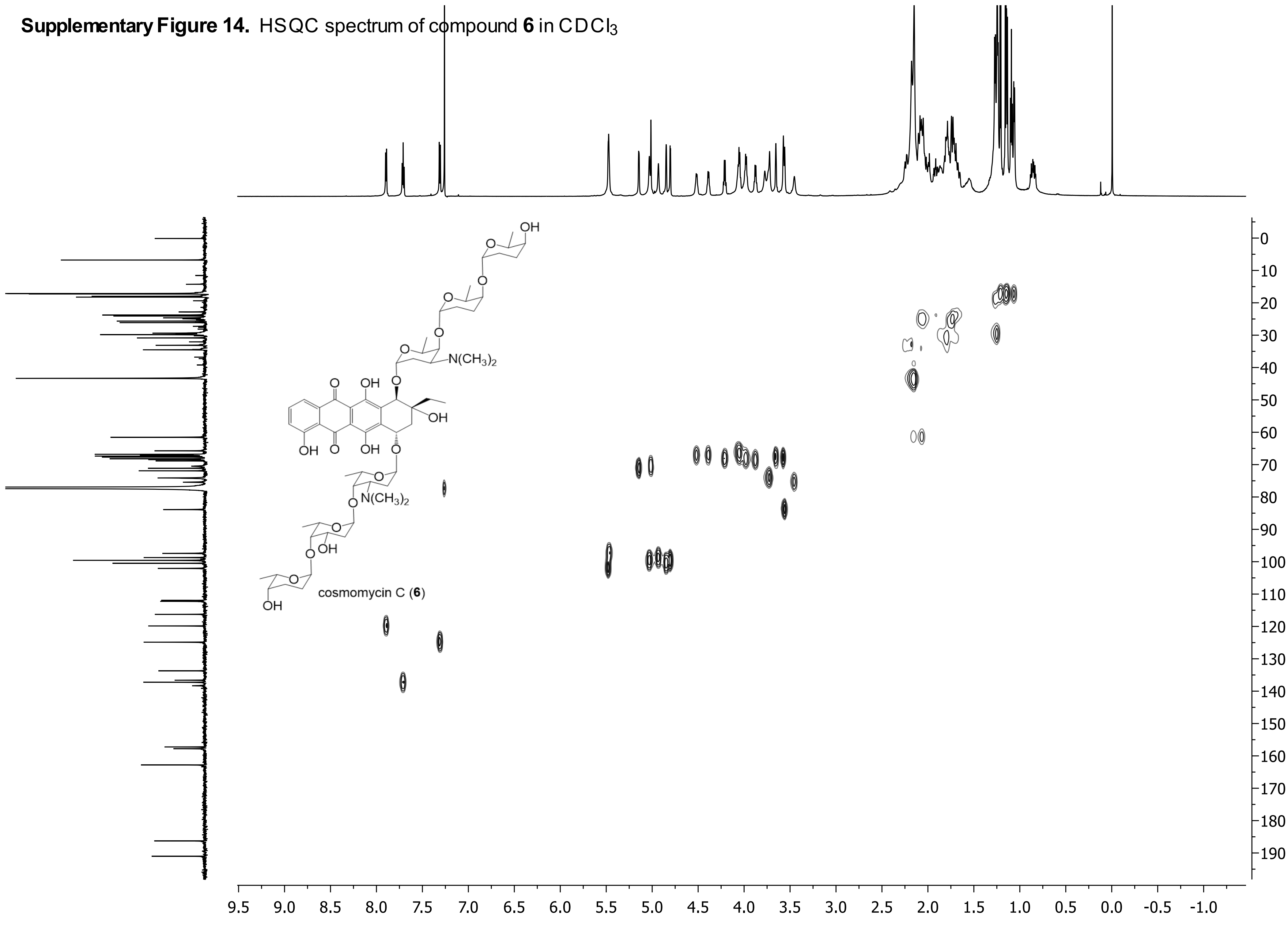

Supplementary Figure 15. HMBC spectrum of compound **6** in $CDCl_3$

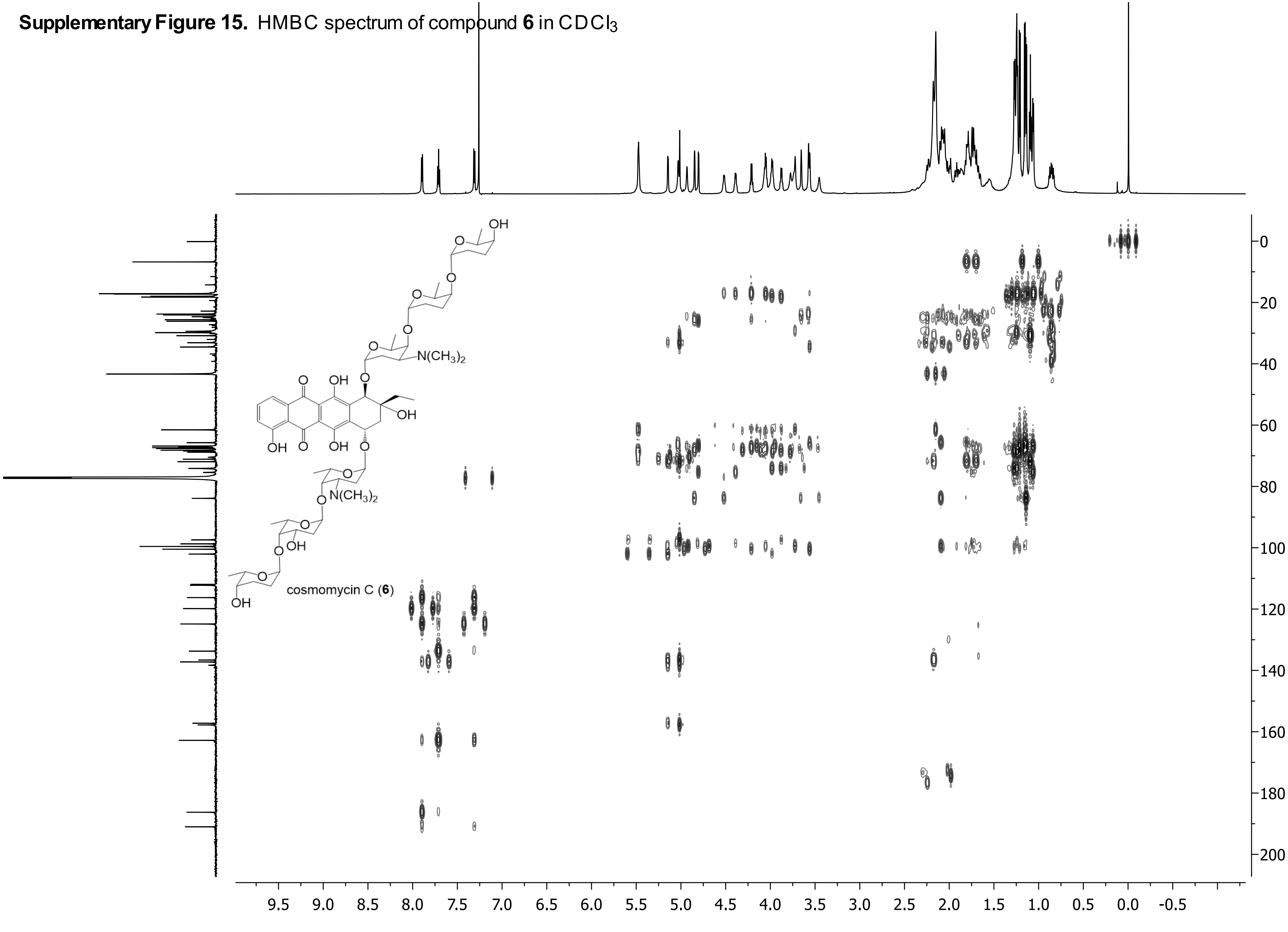

Supplementary Figure 16. ^{1}H NMR (700 MHz) spectrum of compound **7** in $CDCl_3$

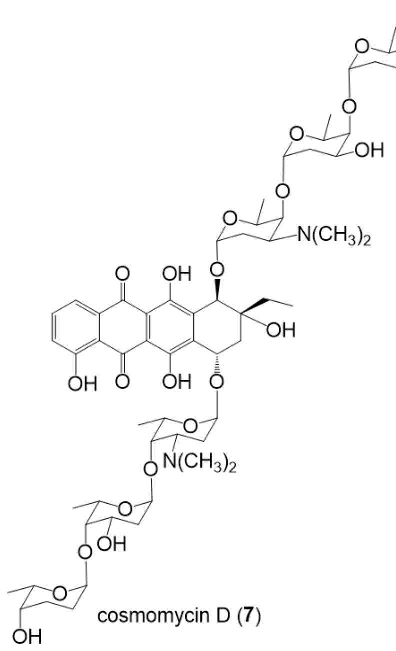

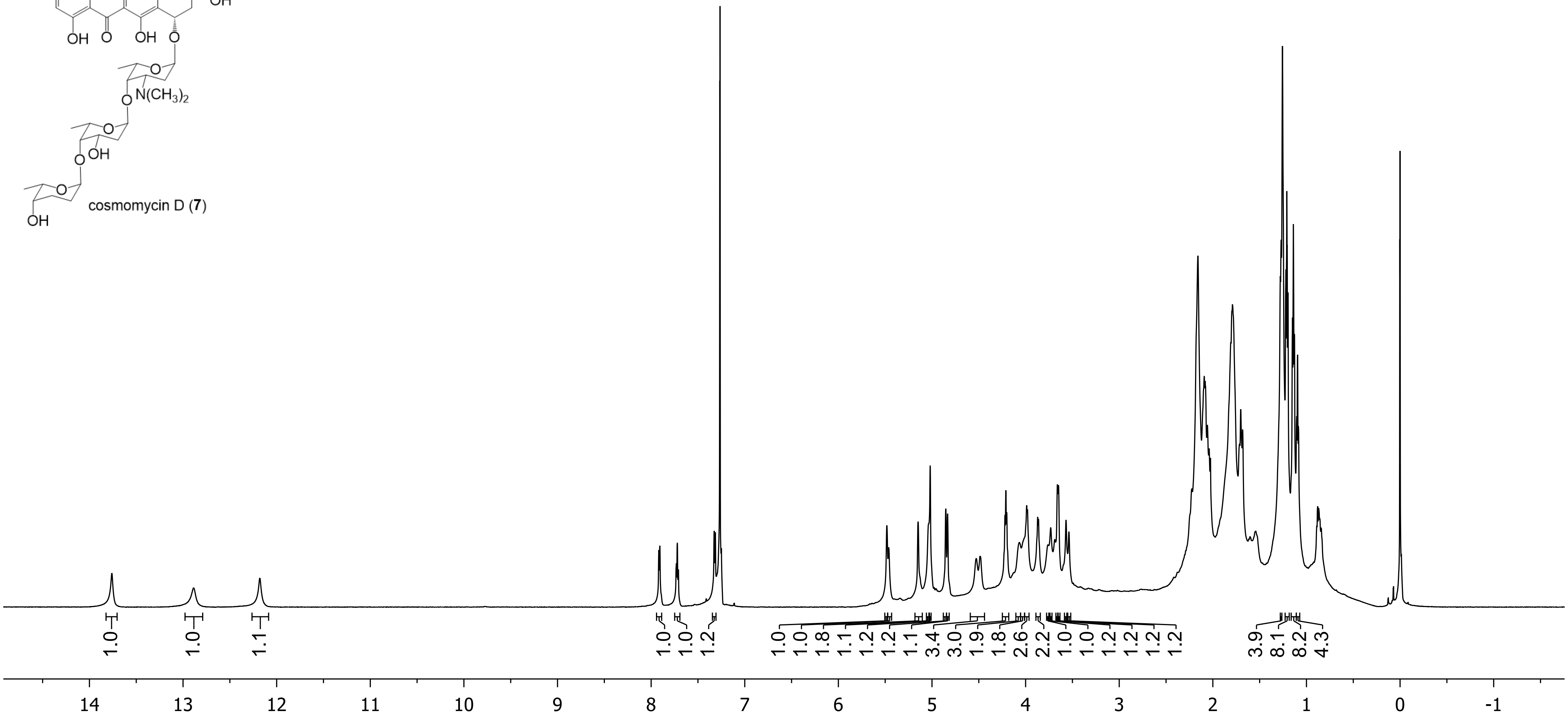

Supplementary Figure 17. ^{13}C NMR (175 MHz) spectrum of compound **7** in $CDCl_3$

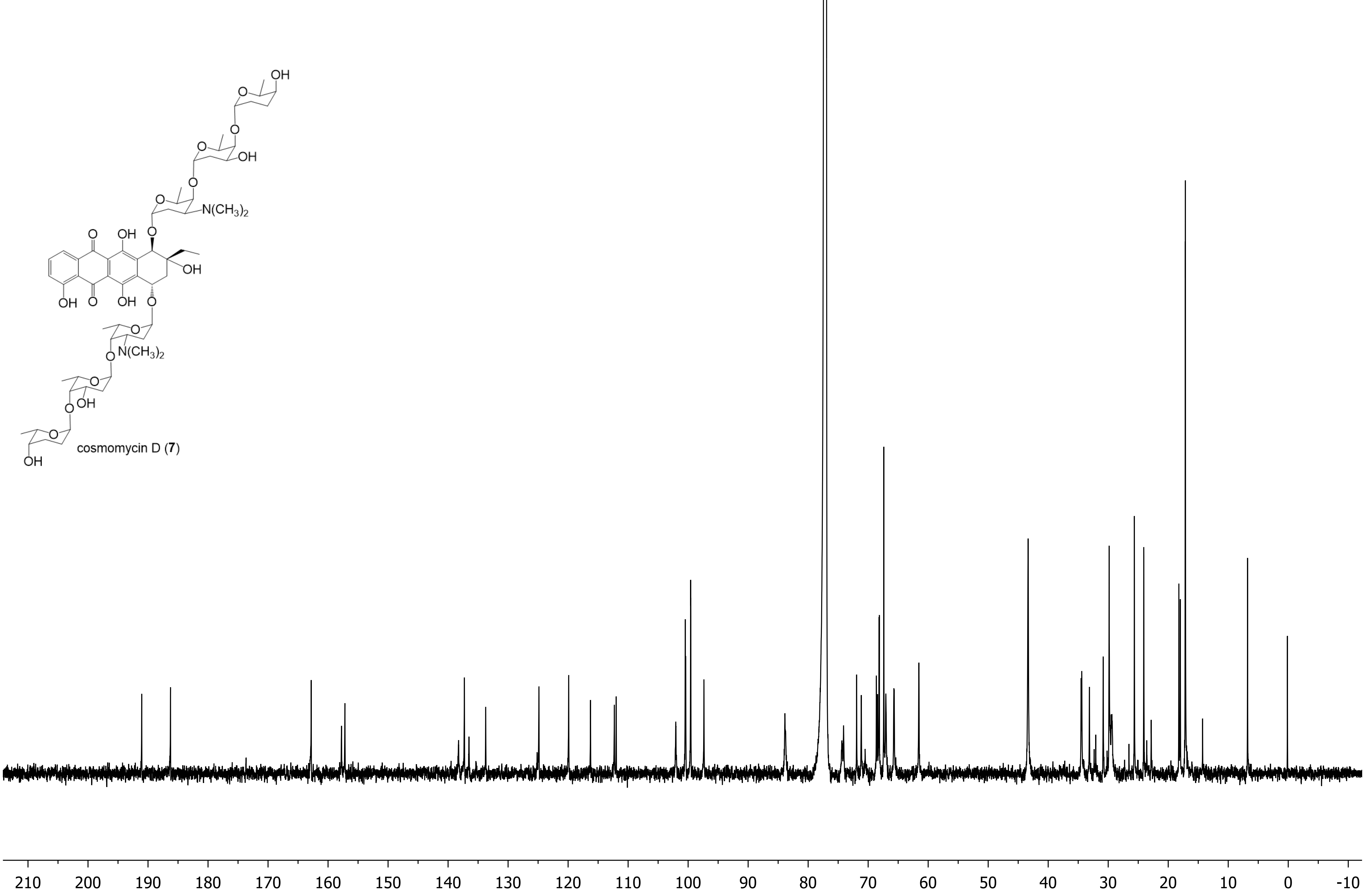

Supplementary Figure 18. DEPT 135 NMR (175 MHz) spectrum of compound **7** in $CDCl_3$

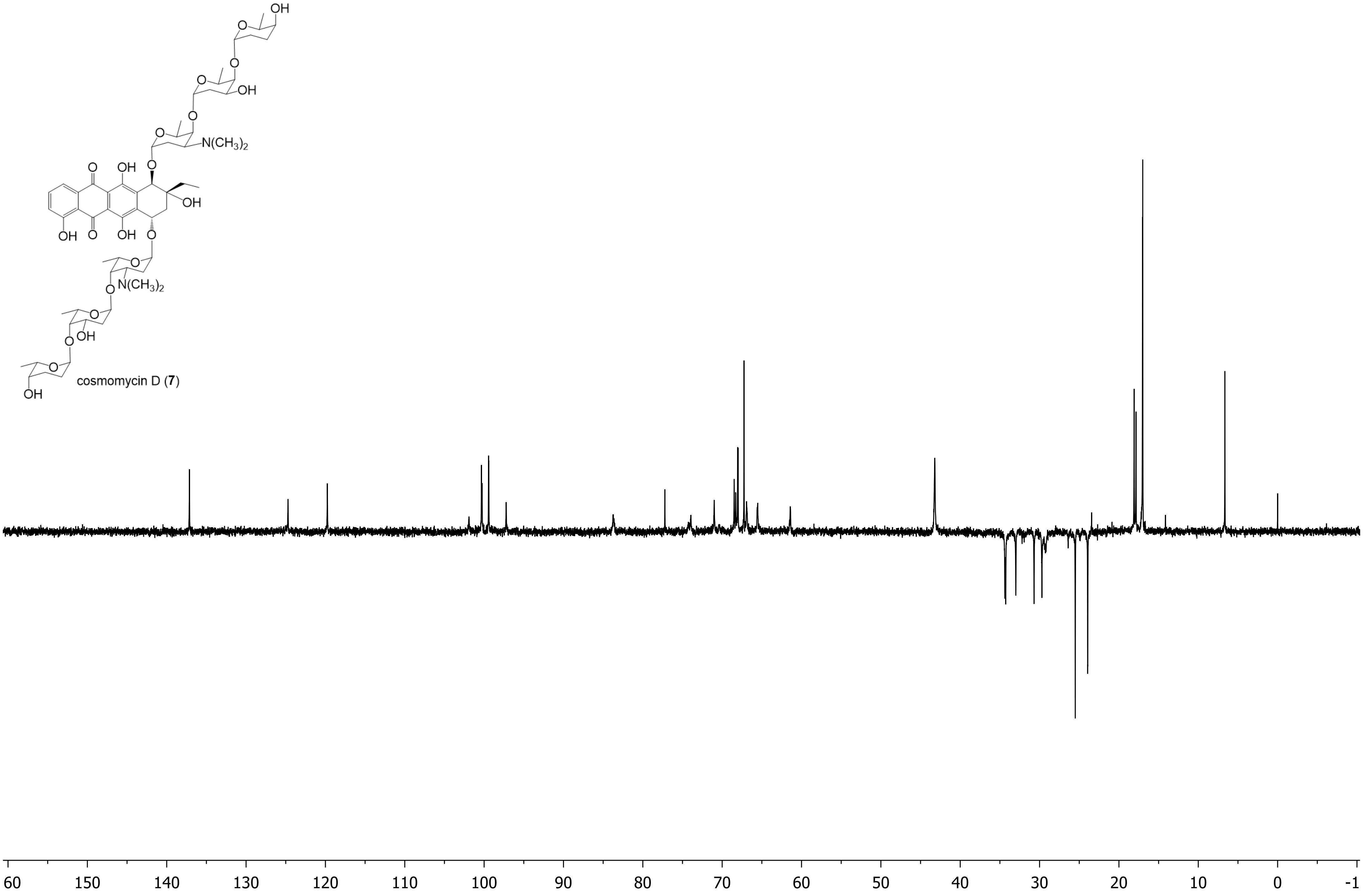

cosmomycin D (**7**)

Supplementary Figure 19. ^{1}H-^{1}H COSY spectrum of compound **7** in $CDCl_3$

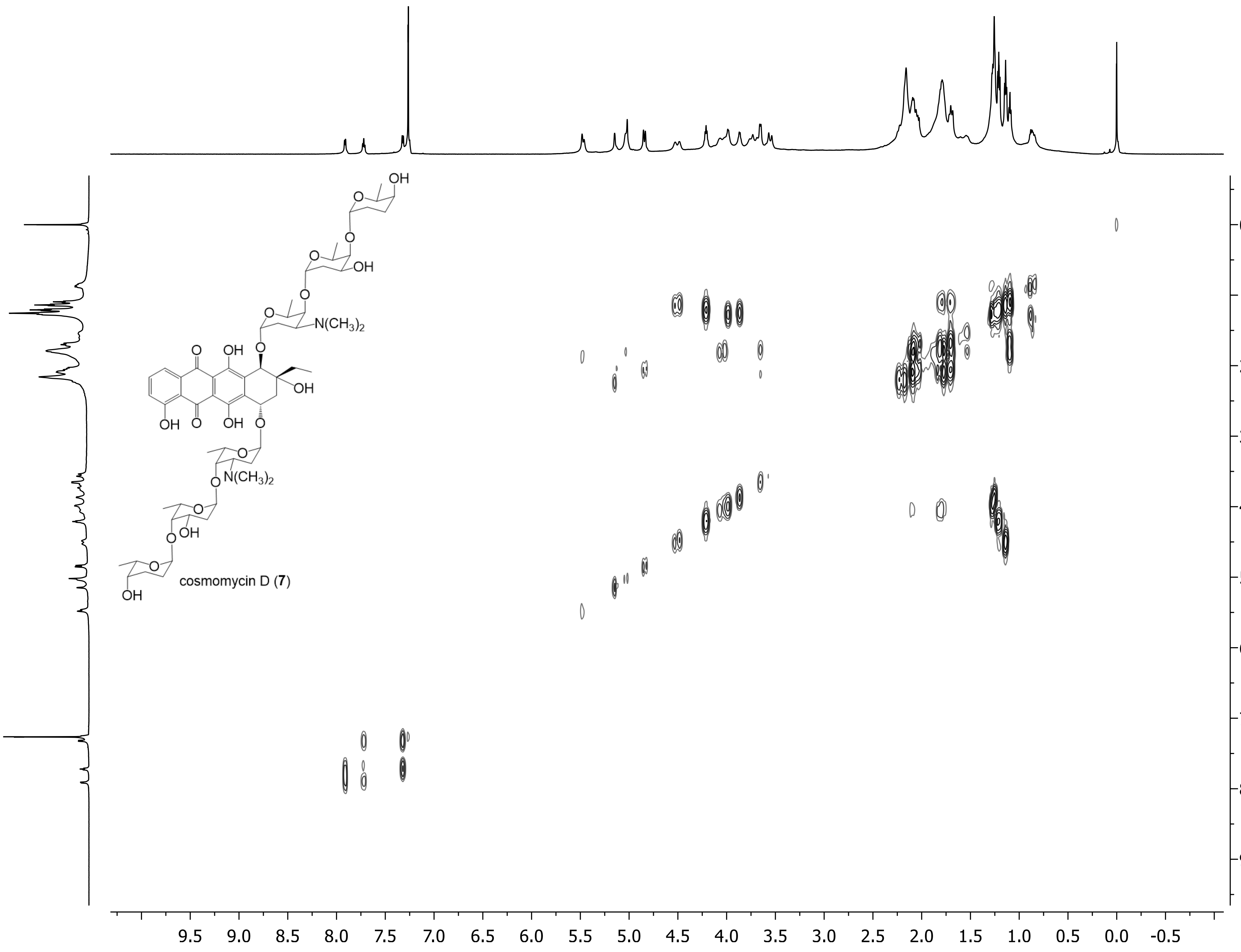

Supplementary Figure 20. HSQC spectrum of compound **7** in $CDCl_3$

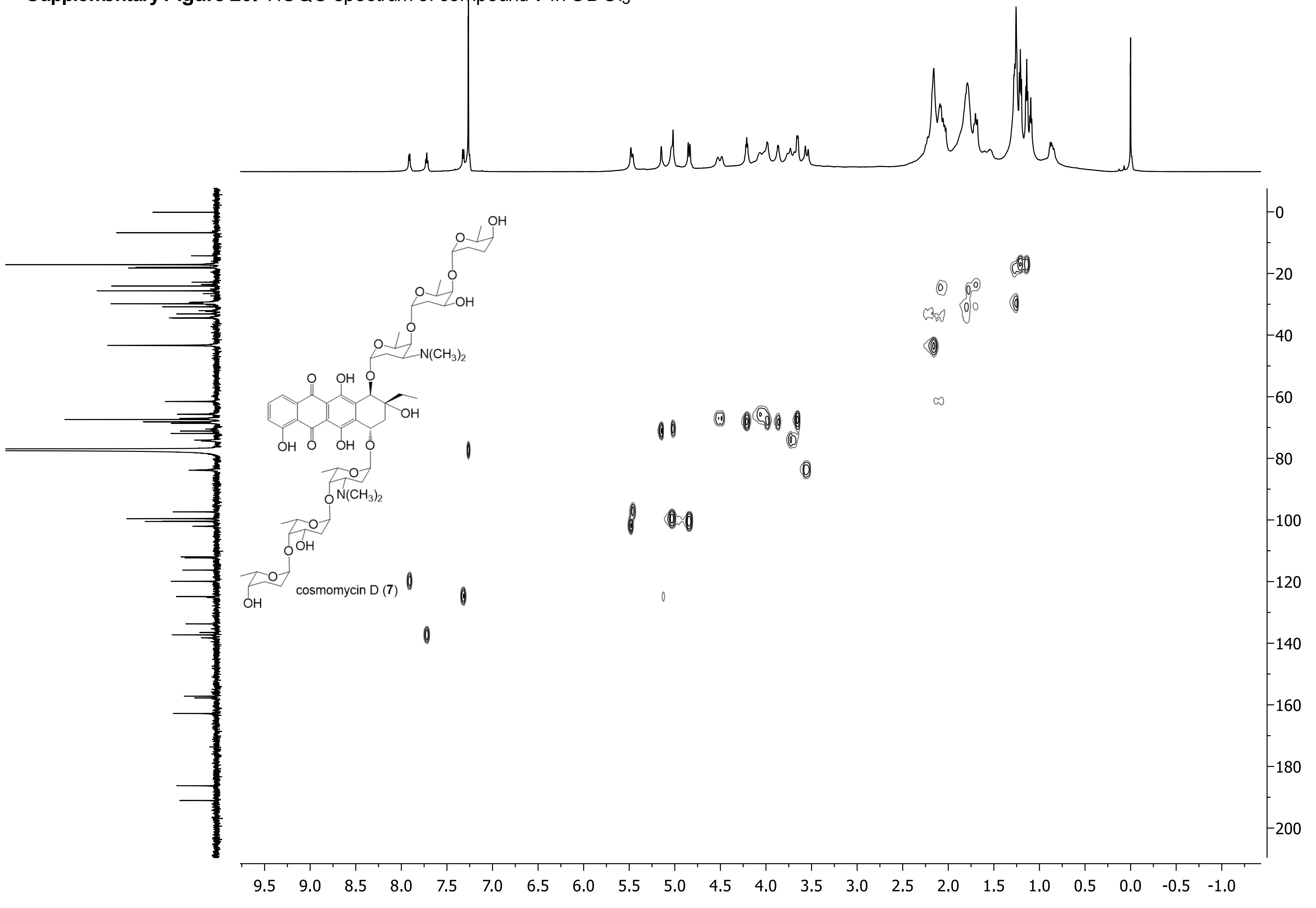

Supplementary Figure 21. HMBC spectrum of compound **7** in $CDCl_3$

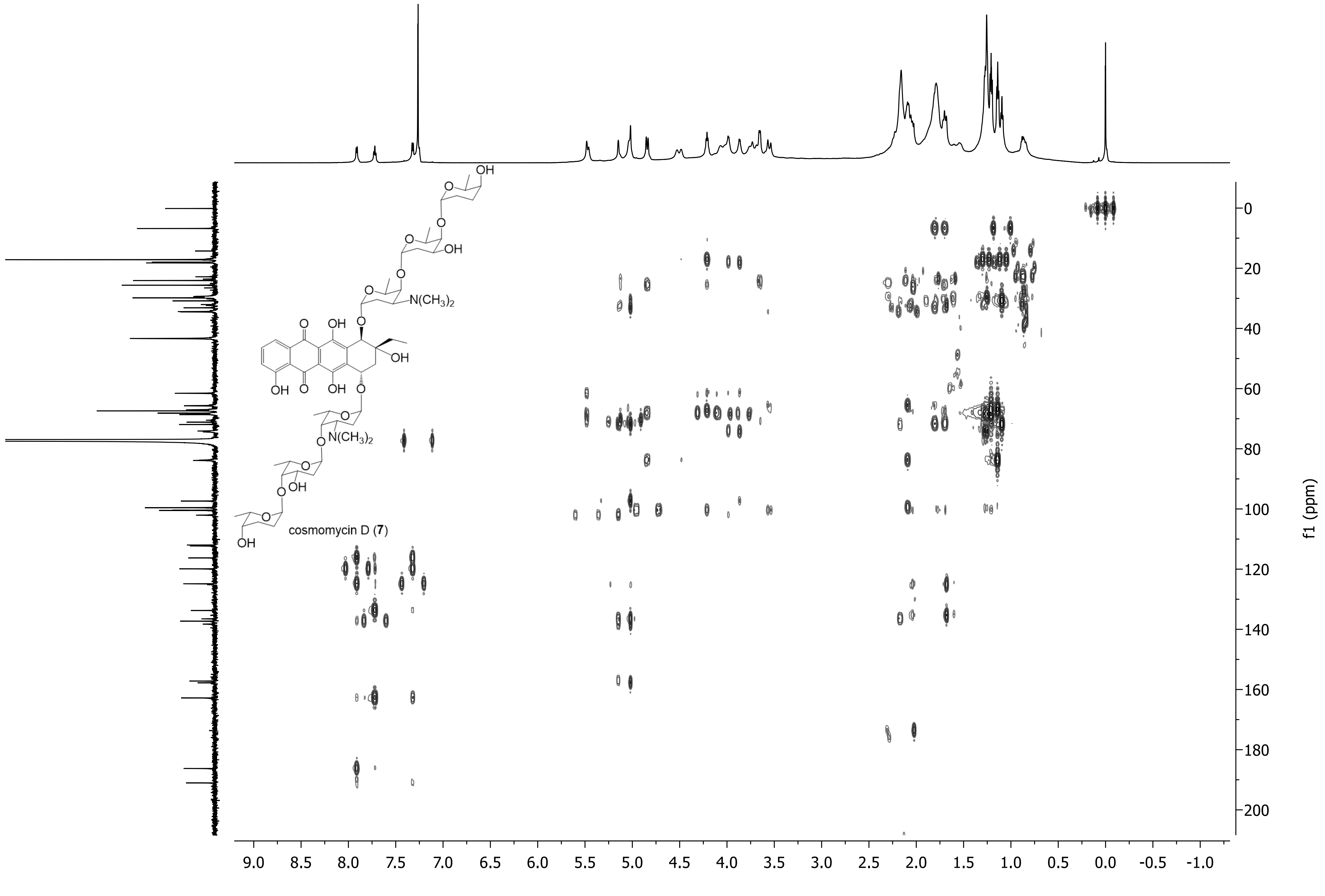

Supplementary Figure 22. ^{1}H NMR (700 MHz) spectrum of compound **8** in $CDCl_3$

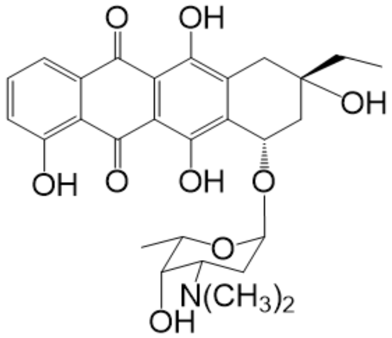

10-decarbomethoxy-ε-rhodomycin(**8**)

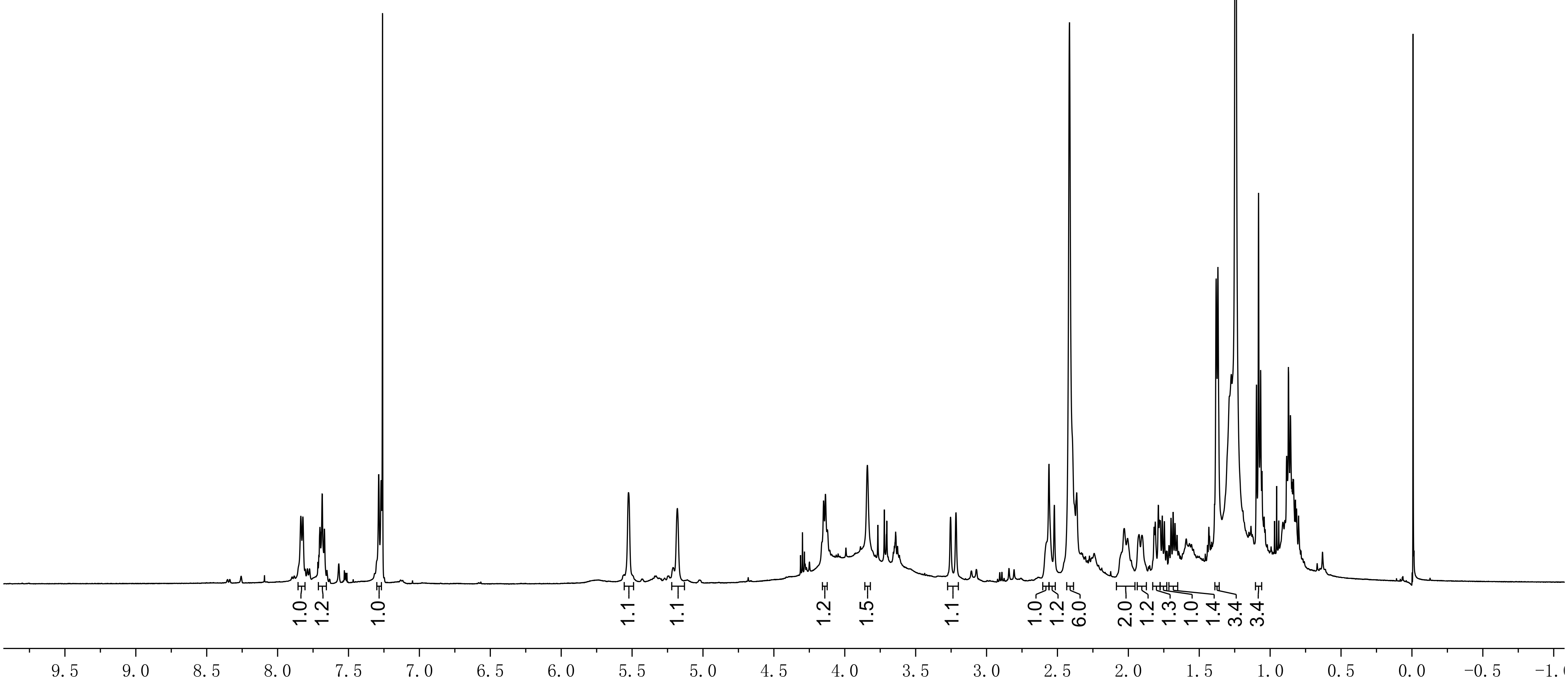

Supplementary Figure 23. ^{13}C NMR (175 MHz) spectrum of compound **8** in $CDCl_3$

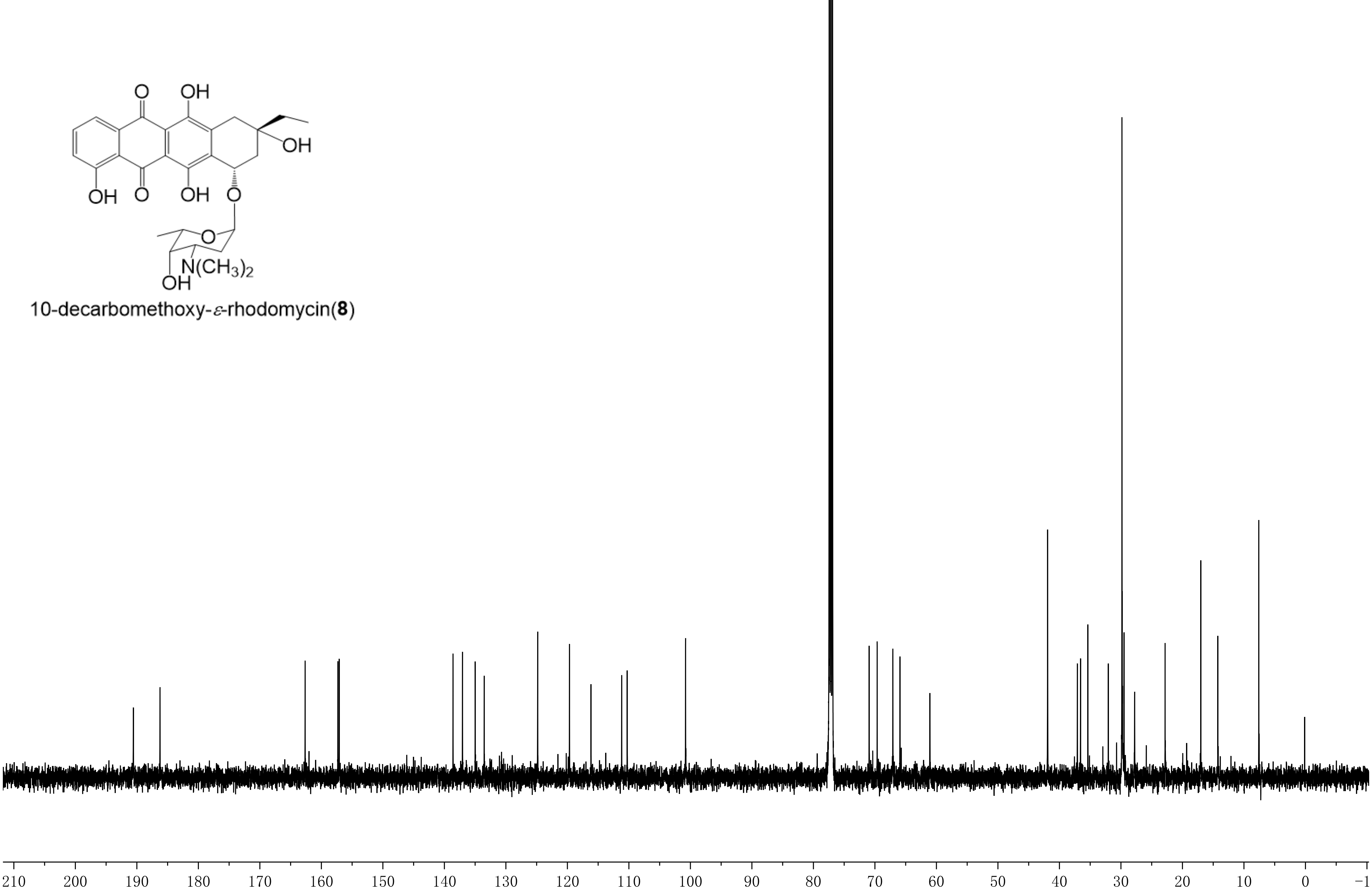

Supplementary Figure 24. DEPT 135 NMR (175 MHz) spectrum of compound **8** in $CDCl_3$

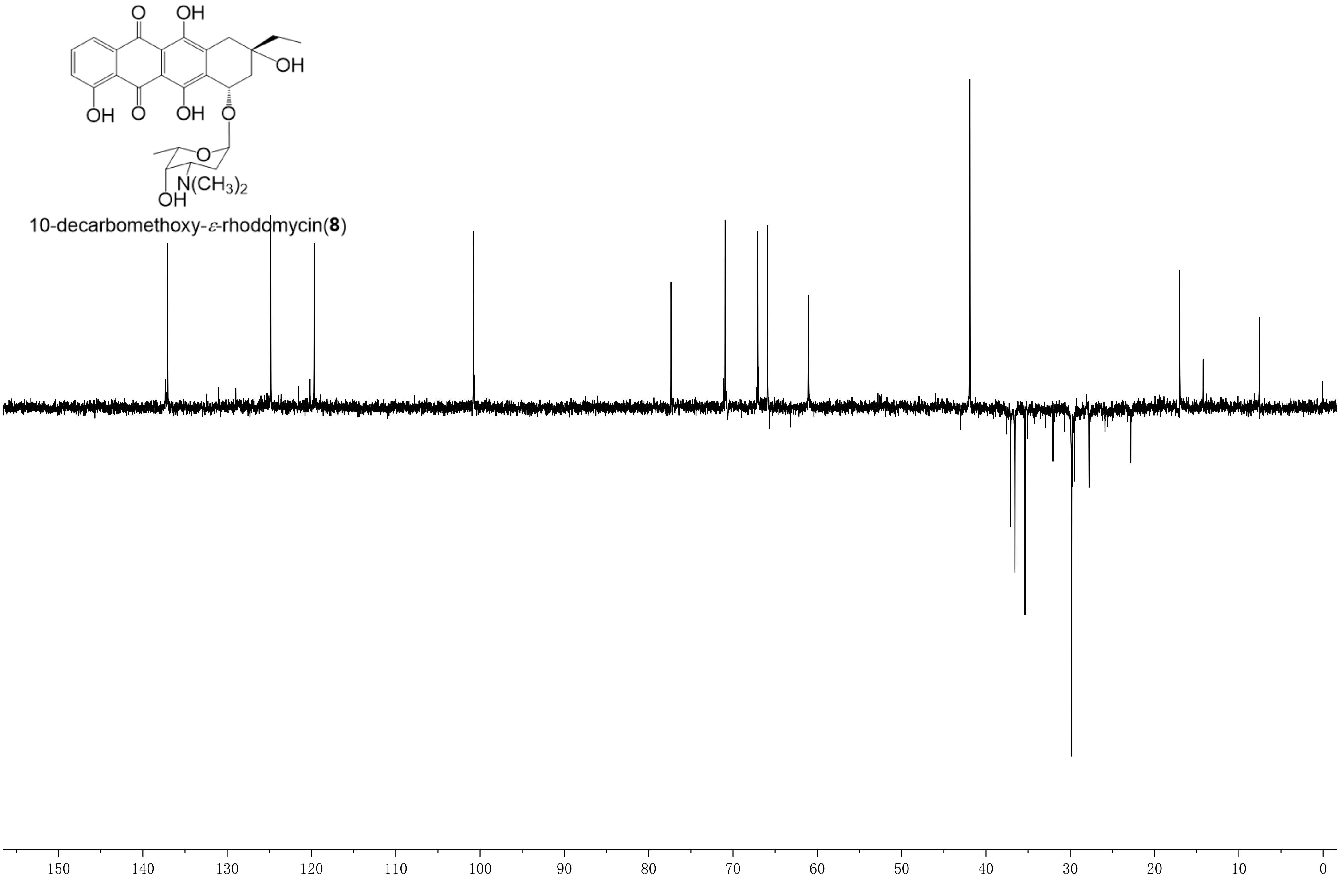

Supplementary Figure 25. ^{1}H-^{1}H COSY spectrum of compound **8** in $CDCl_3$

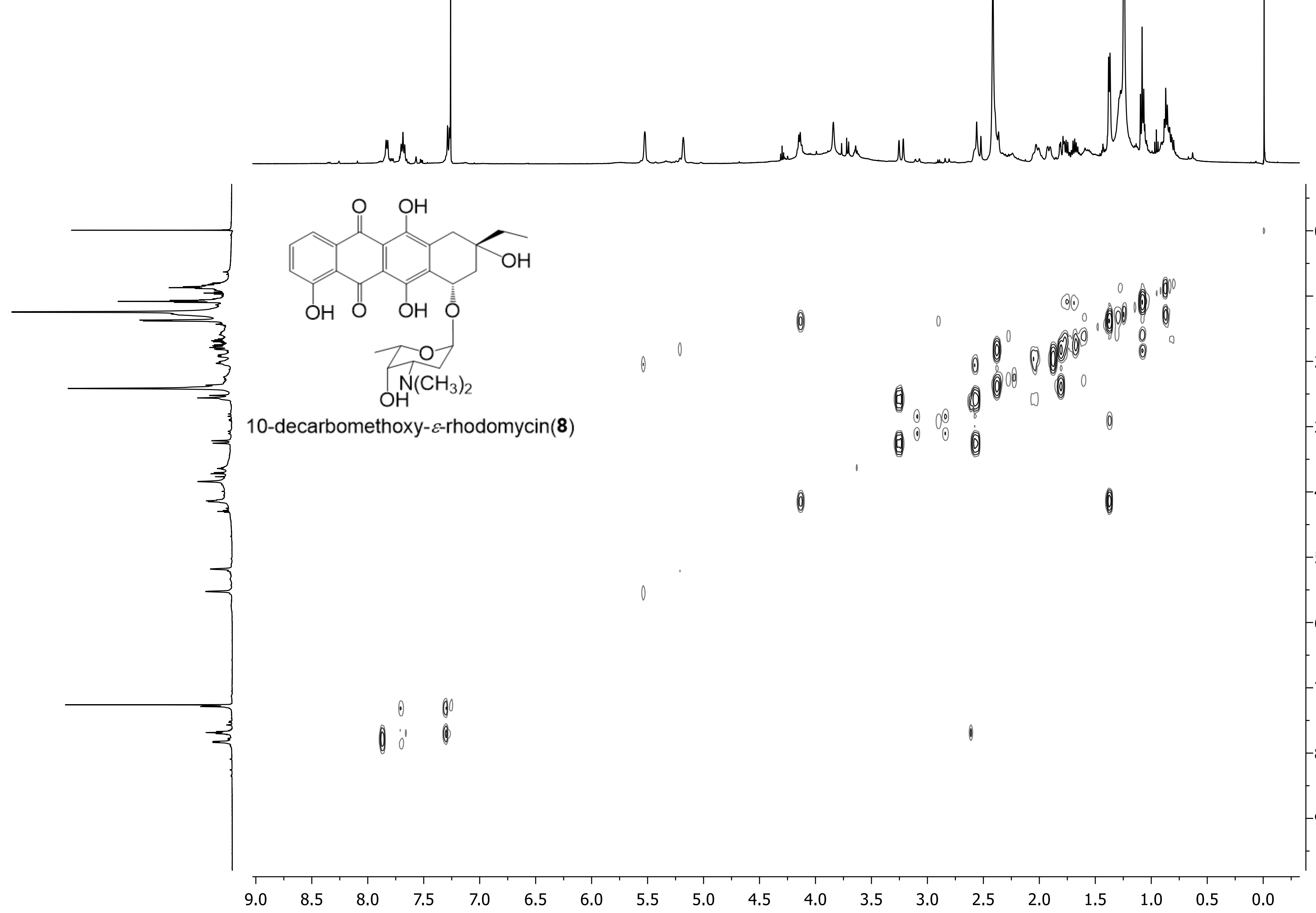

Supplementary Figure 26. HSQC spectrum of compound **8** in $CDCl_3$

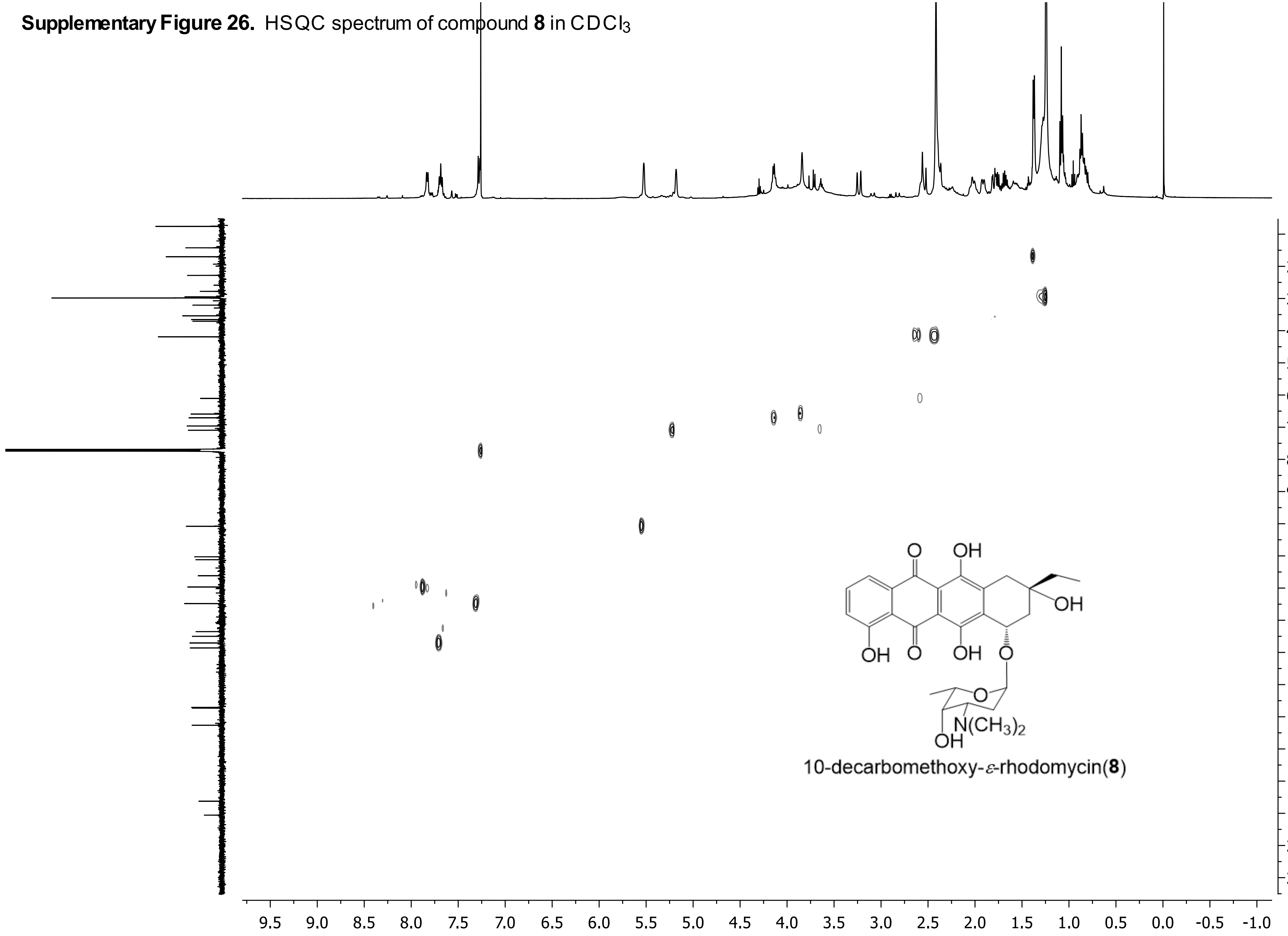

Supplementary Figure 27. HMBC spectrum of compound **8** in $CDCl_3$

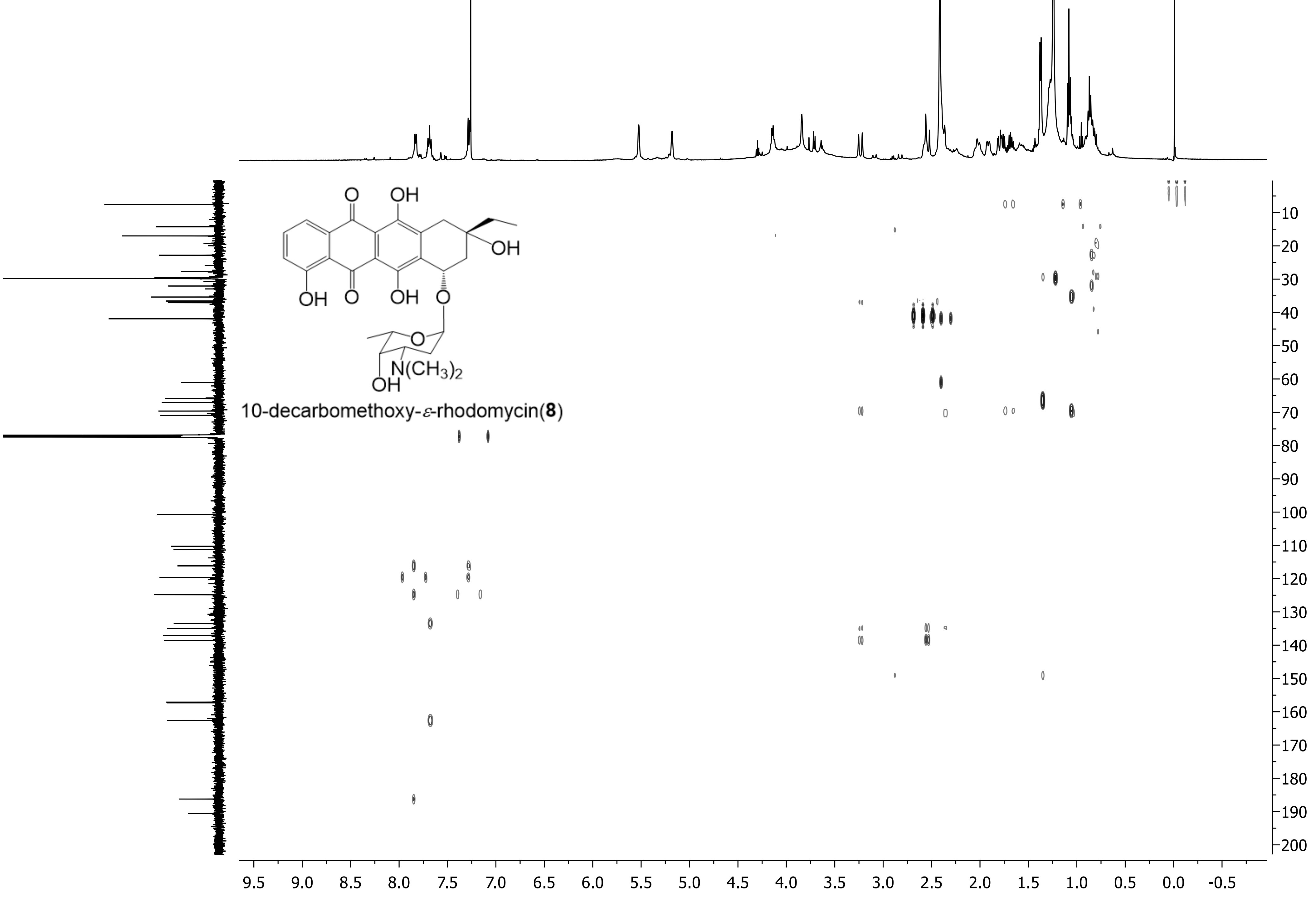

Supplementary Figure 28. ^{1}H NMR (700 MHz) spectrum of compound **9** in $CDCl_3$

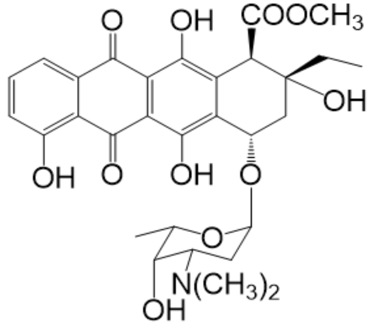

ε-rhodomycin (**9**)

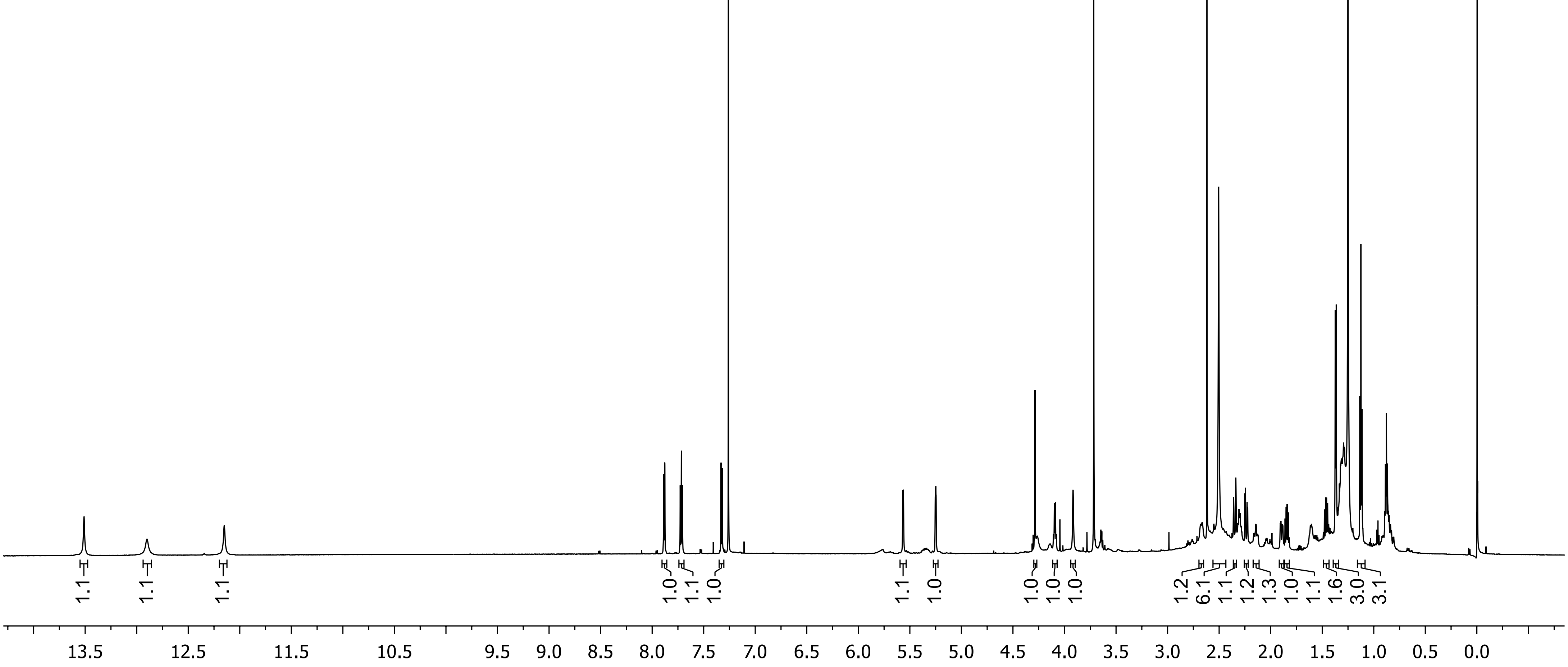

Supplementary Figure 29. ^{13}C NMR (175 MHz) spectrum of compound **9** in $CDCl_3$

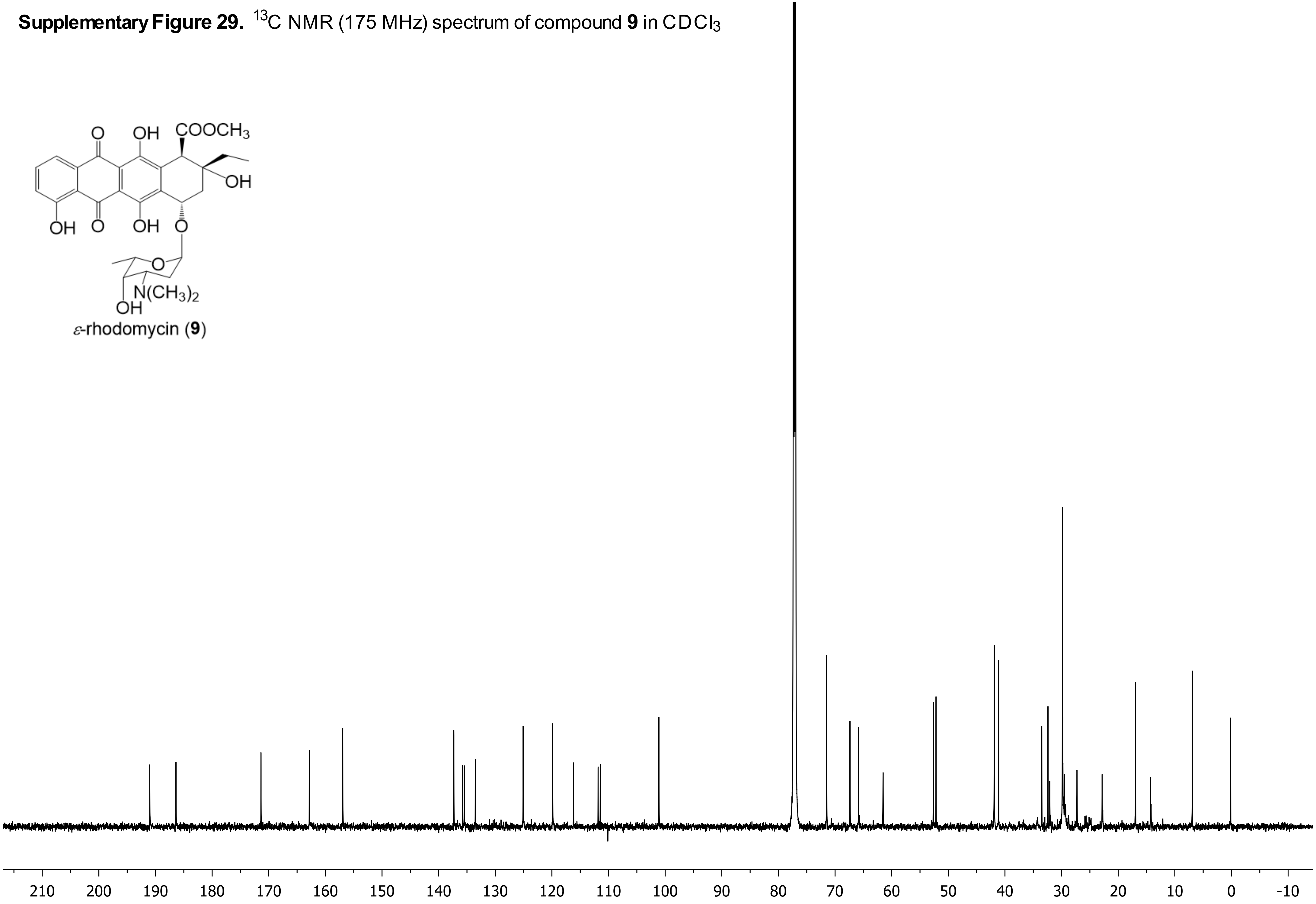

Supplementary Figure 30. DEPT 135 NMR (175 MHz) spectrum of compound **9** in $CDCl_3$

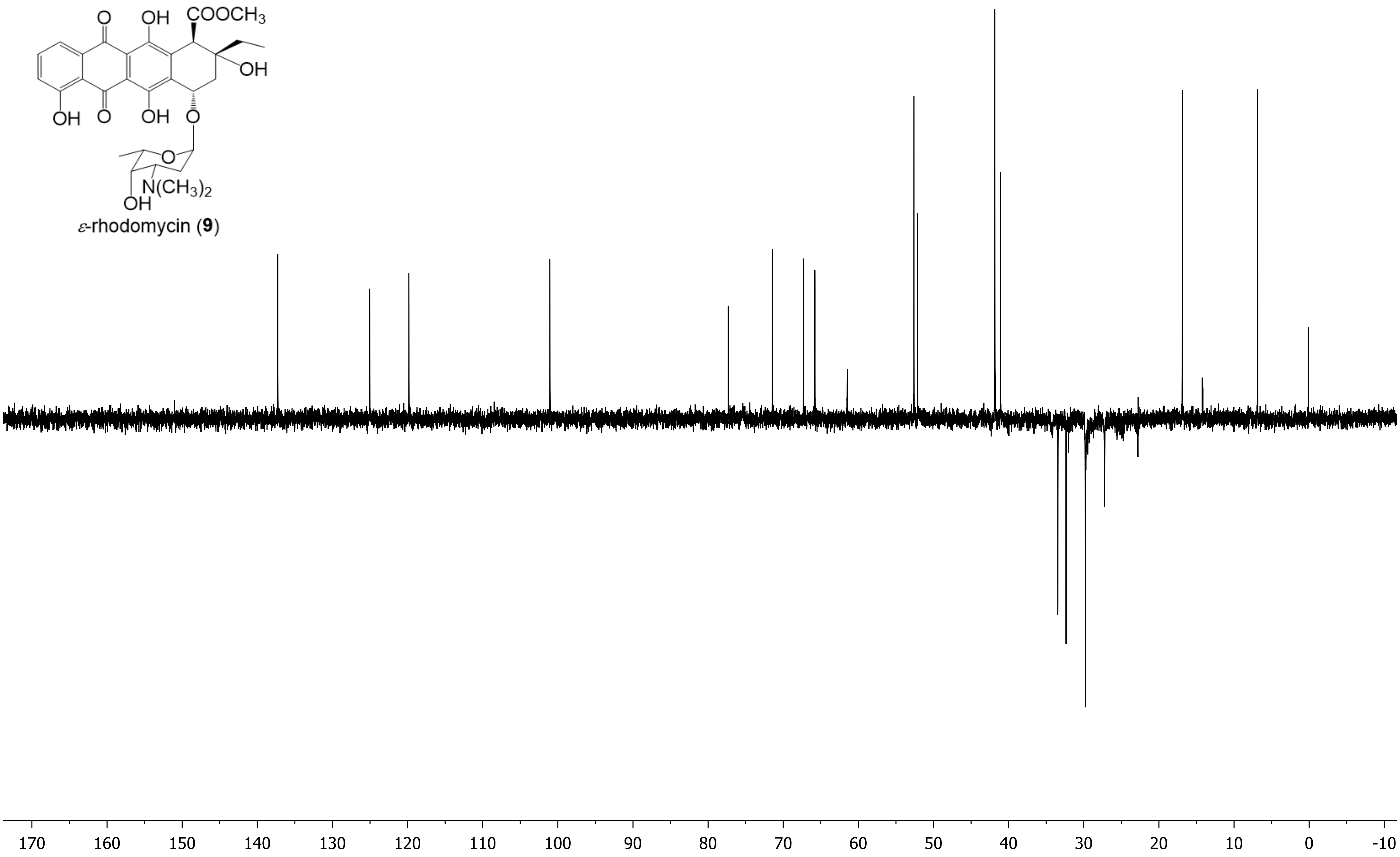

Supplementary Figure 31. ^{1}H-^{1}H COSY spectrum of compound **9** in $CDCl_3$

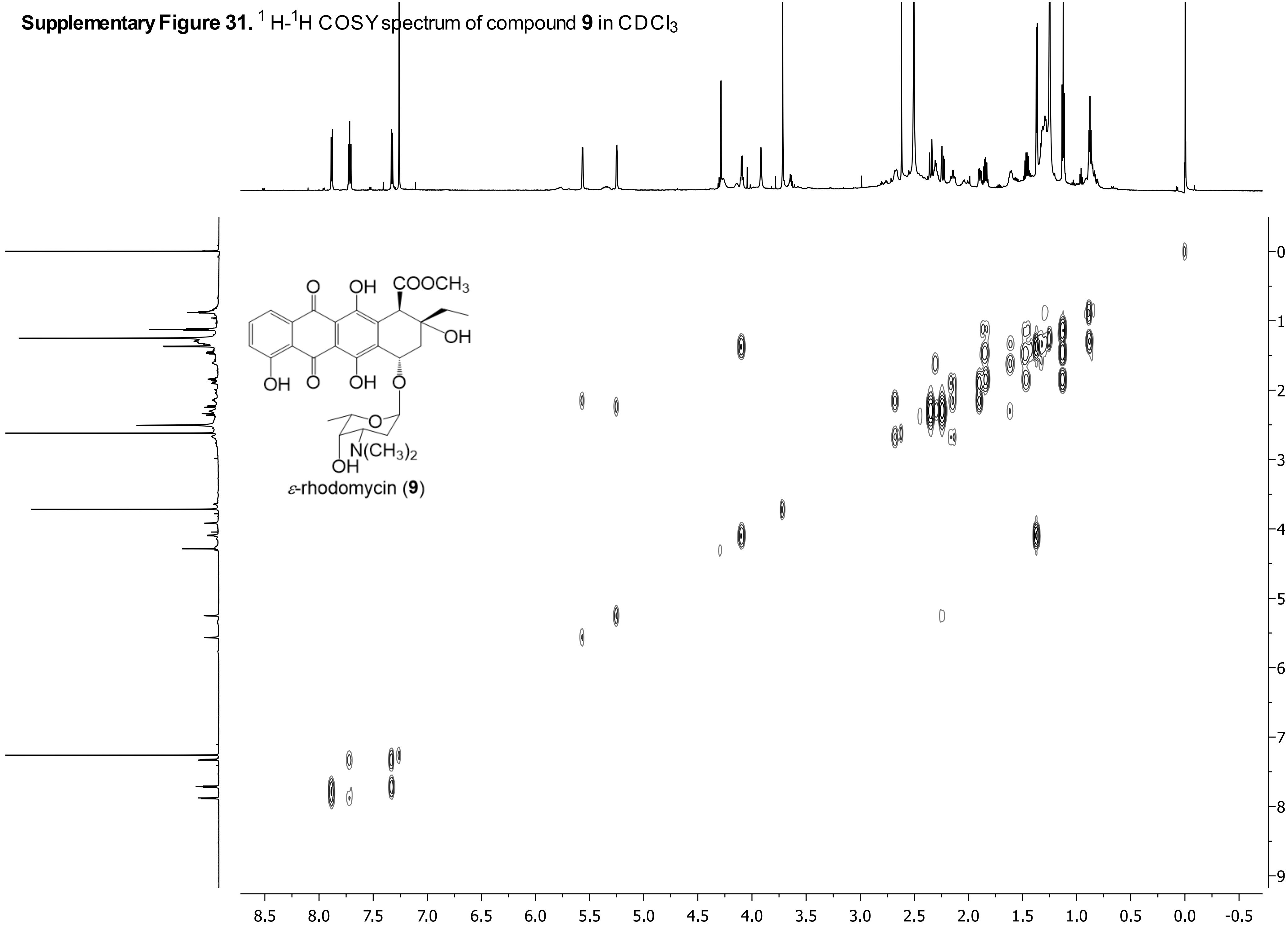

Supplementary Figure 32. HSQC spectrum of compound **9** in $CDCl_3$

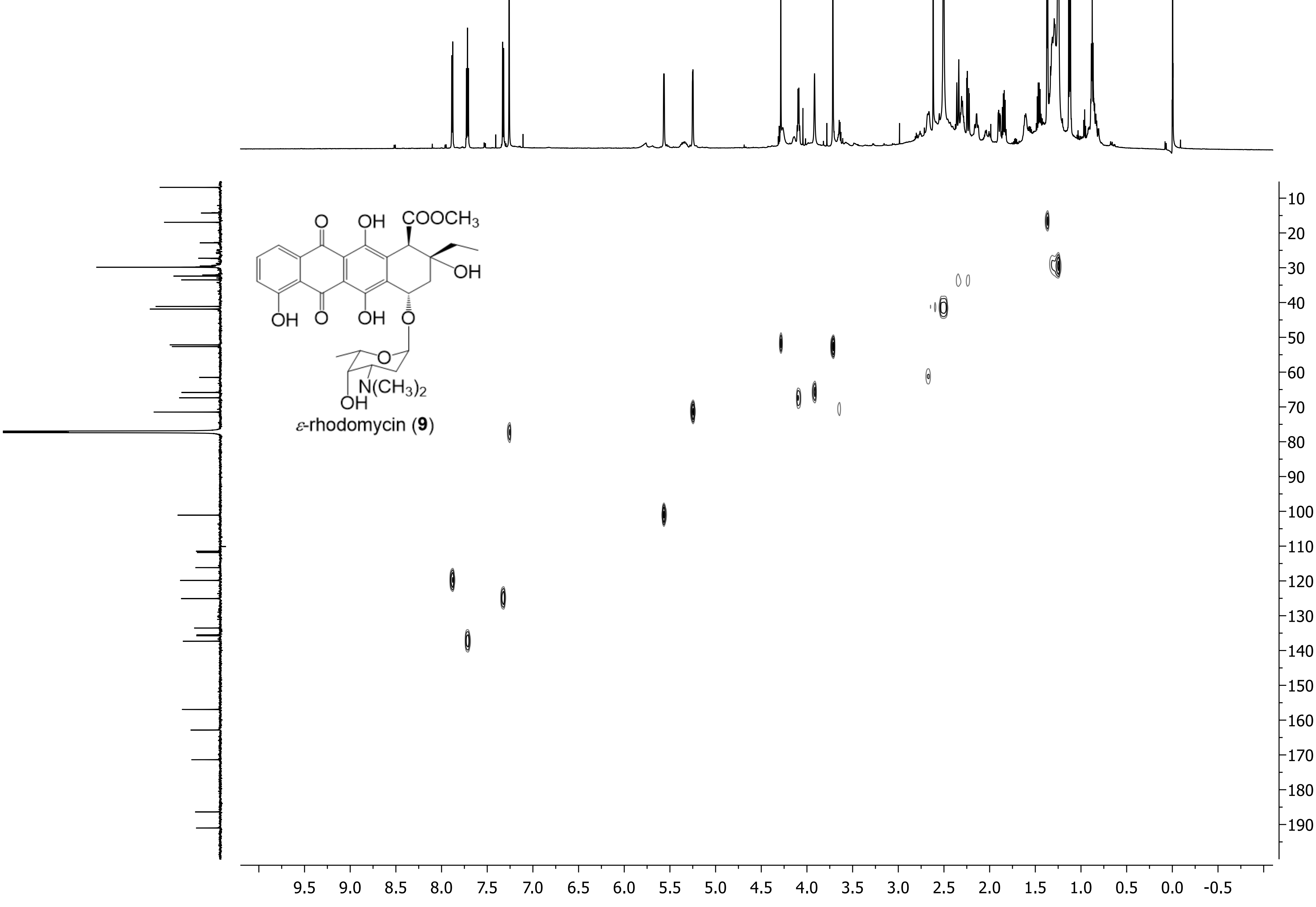

Supplementary Figure 33. HMBC spectrum of compound **9** in $CDCl_3$

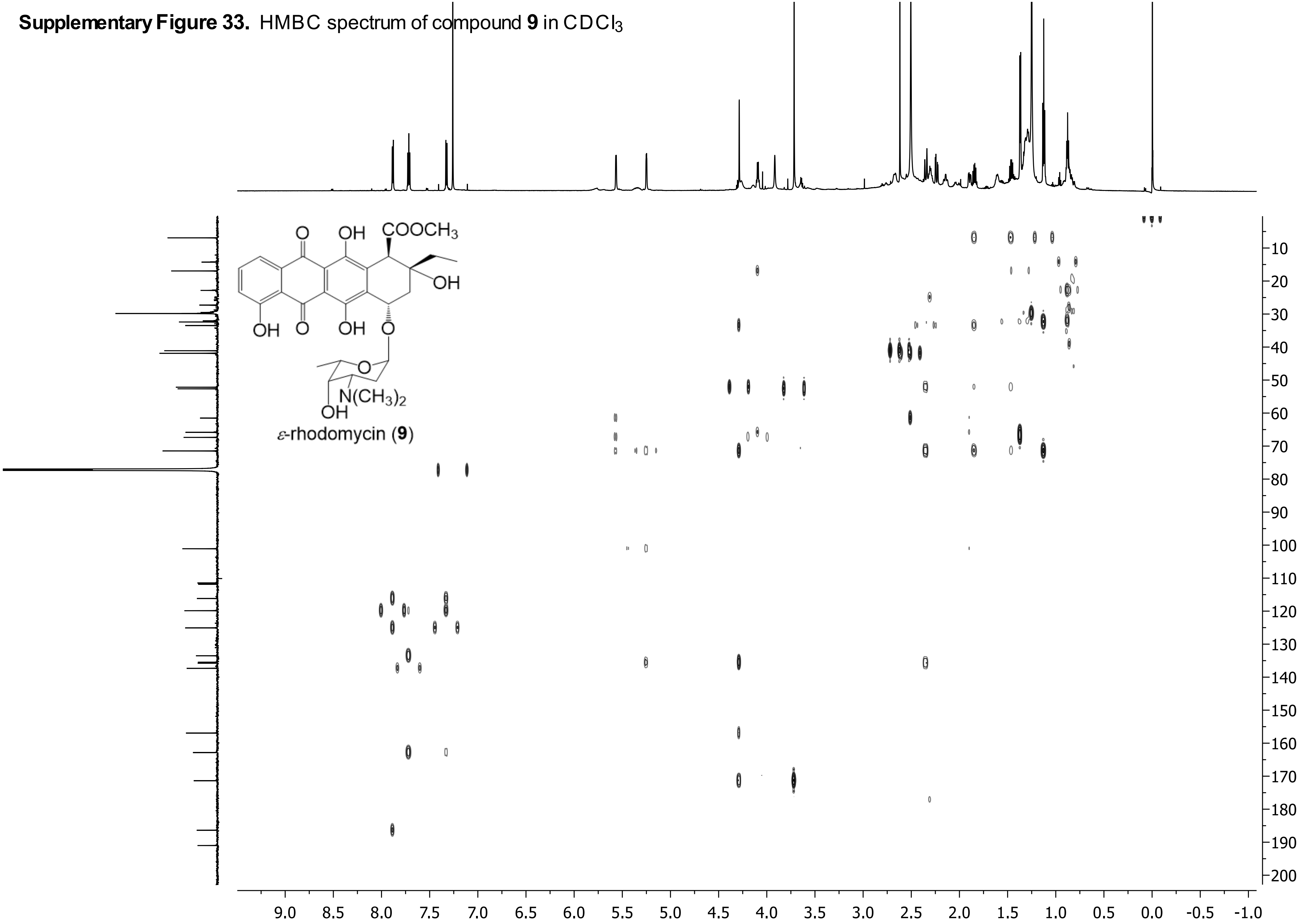

Supplementary Table 1. The gene cluster for biosynthesis of cytorhodin in *Streptomyces* sp. SCSIO 1666 and proposed functions

gene	size[a]	proposed function	*cos* homolog (identity)	other homolog (identity)
orf1	2238	hypothetical protein	—	—
orf2	259	hypothetical protein	—	—
orf3	246	hypothetical protein	—	—
cytR1	296	MarR family transcriptional regulator	—	—
cytA	149	nitroreductase family deazaflavin-dependent oxidoreductase	nitroreductase family deazaflavin-dependent oxidoreductase (93%)	AclJ (64%)
cytB	536	FAD-linked oxidase	FAD-binding oxidoreductase (89%)	AknOx (60%)
cytC	284	aldo/keto reductase	aldo/keto reductase (92%)	—
cytD	291	glucose-1-phosphate thythymidylyltransferase	glucose-1-phosphate thythymidylyltransferase (97%)	NivY (73%)
cytE	84	acyl carrier protein	actinorhodin polyketide synthase (88%)	AknD (56%)
cytR2	295	transcriptional regulator	transcriptional regulator (95%)	AclI (63%)
cytF	285	methyltransferase	methyltransferase (93%)	AknG (66%)
cytG	145	aklanonic acid methyl ester cyclase	ester cyclase (95%)	DnrD (87%)
cytH	367	O-methyltransferase	methyltransferase (89%)	RdmB (84%)
cytI	298	alpha/beta hydrolase, esterase	alpha/beta hydrolase (93%)	RdmC (92%)
cytJ	265	SAM-dependent methyltransferase	SAM-dependent methyltransferase (88%)	SnogX (61%)
cytK	531	FAD-dependent oxidoreductase	FAD-dependent oxidoreductase (94%)	RdmE (91%)
cytS1	346	NDP-hexose-3-ketoreductase	Oxidoreductase (94%)	RdmF (92%)
cytL	376	cytochrome P450 like protein	hypothetical protein (75%)	RdmG (73%)
cytG1	439	glycosyltransferase	glycosyltransferase (95%)	RdmH (92%)
cytS2	433	RfbH/ NDP-hexose 3,4-dehydratase	lipopolysaccharide biosynthesis protein (95%)	RdmI (95%)
cytM	261	ketoacyl reductase	ketoacyl reductase (94%)	RdmJ (94%)
cytN	452	aromatase	actinorhodin polyketide synthase (90%)	RdmK (90%)
cytO	219	C-7 ketoreductase	ketoreductase (91%)	DauE (57%)
cytP	135	hydroxylacyl-CoA dehydrogenase/cyclase	hydroxylacyl-CoA dehydrogenase (87%)	AknV (61%)
cytQ	259	cyclase	cyclase (95%)	StfY (71%)
cytR	242	SAM-dependent methyltransferase	SAM-dependent methyltransferase (95%)	Niv4 (68%)
cytS	317	NDP-hexose aminotransferase	DnrJ aminotransferase (95%)	—
cytT1	279	transposase	—	—

cytT2	48	toxin-antitoxin system, antitoxin	—	—
cytS3	485	NDP-hexose 2,3-dehydratase	NDP-hexose 2,3-dehydratase (88%)	SnogH (60%)
cytS4	345	NAD-dependent epimerase	NAD(P)-dependent oxidoreductase (83%)	NivM (60%)
cytS5	208	dTDP-4-dehydrorhamnose 3,5-epimerase	CosL (89%)	NivL (63%)
cytG2	430	glycosyl transferase family 28	CosK (95%)	AknK (65%)
cytR3	187	PadR-like family transcriptional regulator	CosS (95%)	AclS (53%)
cytU	326	ornithine cyclodeaminase	CosY (80%)	—
cytV	111	antibiotic biosynthesis monooxygenase	CosX (92%)	SnoaB (56%)
cyt1	422	beta-ACP synthase	CosB (94%)	AknB (71%)
cyt2	414	beta-ketoacyl synthase	CosC (89%)	AknC (66%)
cyt3	344	3-oxoacyl-ACP synthase	CosE (91%)	AknE2 (65%)
cyt4	350	polyketide synthase	CosF (83%)	AknF (66%)
cytW	416	cytochrome P450 like protein	CosT (83%)	AknT (45%)
cytG3	426	glycosyl transferase family 28	CosG (94%)	DnrS (59%)
cytS6	327	dTDP-glucose 4,6-dehydratase	CosH (93%)	Niv1 (75%)
cytT3	328	ABC transportor	—	—
cytT4	276	ABC-2 type transporter	multidrug ABC transporter	—
			permease (97%)	—
cytX	174	glutathione peroxidase	glutathione peroxidase (87%)	—
cytT5	761	ABC transportor	excinuclease ABC subunit UvrA (95%)	—
cytY	182	DinB family protein	hypothetical protein DF19_01985	—
cytR4	200	PadR family transcriptional regulator	transcriptional regulator	—
orf(-1)	91	hypothetical protein	membrane protein	—
orf(-2)	475	Ribonuclease BN	Ribonuclease BN	—

[a]Size in units of amino acids (aa); *cos*: biosynthetic gene cluster of cosmomycin D in *Streptomyces olindensis DAUFPE 5622*; ABC: ATP-binding cassete.

Supplementary Table 2. ^{1}H (700 MHz) and ^{13}C NMR (175 MHz) spectral data of cosmomycin C (6) and cosmomycin D (7) in $CDCl_3$.

	6		A262-2 (cosmomycin C)[1]	7		A262-3 (cosmomycin D)[1]
	δ_C	δ_H	δ_C	δ_C	δ_H	δ_C
1	119.9, CH	7.90, d (7.5)	119.7, CH	119.9, CH	7.91, d (7.3)	119.7, CH
2	137.2, CH	7.71, t (7.9)	137, CH	137.3, CH	7.72, t (7.4)	137.1, CH
3	124.9, CH	7.31, d (8.4)	124.7, CH	124.9, CH	7.32, d (8.4)	124.6, CH
4	162.8, C		162.5, C	162.8, C		162.5, C
4a	116.3, C		116.0, C	116.3, C		116.0, C
5	191.0, C		190.7, C	191.1, C		190.7, C
5a	112.3, C		112.0, C	112.3, C		112.0, C
6	157.2, C		157.1, C	157.2, C		157.1, C
6a	136.6, C		136.5, C	136.5, C		136.4, C
7	71.1, CH	5.15, brs	70.9, CH	71.1, CH	5.15, brs	70.9, CH
8	33.1, CH_2	2.24, m; 2.07, m	33.0, CH_2	33.1, CH_2	2.22, d (16.1)	33.0, CH_2
9	71.9, C		71.7, C	71.9, C		71.7, C
10	70.5, CH	5.01, s	70.3, CH	70.5, CH	5.02, brs	70.3, CH
10a	138.3, C		138.2, C	138.3, C		138.2, C
11	157.8, C		157.6, C	157.8, C		157.6, C
11a	111.9 C		111.7, C	112.0, C		111.7, C
12	186.3, C		185.9, C	186.3, C		185.9, C
12a	133.7, C		133.5, C	133.7, C		133.5, C
13	30.9, CH_2	1.80, m	30.7, CH_2	30.8, CH_2	1.5-2.2, m	30.6, CH_2
14	6.8, CH_3	1.09, t (7.4)	6.6, CH_3	6.8, CH_3	1.10, t (6.7)	6.6, CH_3
Sugar moiety attached to C-7						
1'	102.1, CH	5.48, brs	102, CH	102.1, CH	5.48, brs	101.9, CH
2'	29.4, CH_2	1.25, m	29.3, CH_2	29.5, CH_2	1.5-2.2, m	29.2, CH_2
3'	61.6, CH	2.11, m	61.4, CH	61.6, CH	2.11, m	61.3, CH
4'	74.1, CH	3.73, brs	74.1, CH	74.3, CH	3.73, brs	74.2, CH
5'	68.5, CH	3.88, q (6.2)	68.3, CH	68.4, CH	4.21, q (7.3)	68.3, CH
6'	18.0, CH_3	1.24, m	17.8, CH_3	18.0, CH_3	1.21, m	17.8, CH_3
3'-$N(CH_3)_2$	43.3, CH_3	2.15, s	43.3, CH_3	43.3, CH_3	2.16, s	43.2, CH_3
1''	99.6, CH	5.03, brs	99.5, CH	99.6, CH	5.02, brs	99.4, CH
2''	34.5, CH_2	2.18, m	34.4, CH_2	34.5, CH_2	1.5-2.2, m	34.3, CH_2
3''	65.7, CH	4.05, m	65.6, CH	65.7, CH	4.04, m	65.5, CH
4''	83.9, CH	3.56, s	83.7, CH	83.9, CH	3.57, m	83.7, CH
5''	66.8, CH	4.05, q (6.6)	66.7, CH	67.1, CH	4.52, q (6.4)	66.8, CH
6''	17.2, CH_3	1.15, d (6.6)	16.9, CH_3	17.1, CH_3	1.14, m	16.9, CH_3
1'''	100.5, CH	4.85, brs	100.3, CH	100.5, CH	4.85, brs	100.3, CH
2'''	24.1, CH_2	1.5-2.2, m	23.9, CH_2	24.1, CH_2	1.5-2.2, m	23.9, CH_2
3'''	25.6, CH_2	1.5-2.2, m	25.5, CH_2	25.6, CH_2	1.5-2.2, m	25.5, CH_2
4'''	67.4, CH	3.65, brs	67.2, CH	67.4, CH	3.65, brs	67.2, CH
5'''	68.2, CH	3.98, q (6.4)	68.1, CH	68.2, CH	3.97, q (5.6)	68.1, CH
6'''	17.3, CH_3	1.27, d (6.4)	17.1, CH_3	17.2, CH_3	1.14, m	17.1, CH_3
Sugar moiety attached to C-10						
1'	97.4, CH	5.46, brs	97.3, CH	97.4, CH	5.46, brs	97.2, CH
2'	29.8, CH_2	1.5-2.2, m	29.7, CH_2	29.8, CH_2	1.5-2.2, m	29.6, CH_2
3'	61.6, CH	2.11, m	61.4, CH	61.6, CH	2.11, m	61.4, CH
4'	74.2, CH	3.72, brs	74.3, CH	74.4, CH	3.73, brs	74.3, CH
5'	68.8, CH	4.21, q (6.5)	68.7, CH	68.6, CH	4.02, q (6.4)	68.5, CH
6'	18.2, CH_3	1.21, d (6.5)	18.0, CH_3	18.2, CH_3	1.28, m	18.0, CH_3
3'-$N(CH_3)_2$	43.3, CH_3	2.15, s	43.2, CH_3	43.3, CH_3	2.15, s	43.2, CH3
1''	98.7, CH	4.93, brs	98.6, CH	99.6, CH	4.83, brs	99.4, CH
2''	24.7, CH_2	1.5-2.2, m	24.5, CH_2	34.4, CH_2	1.5-2.2, m	34.2, CH_2
3''	24.9, CH_2	1.5-2.2, m	24.8, CH	65.7, CH	4.04, m	65.6, CH
4''	75.4, CH	3.45, brs	75.3, CH	83.7, CH	3.54, m	83.5, CH
5''	67.0, CH	4.51, q (6.4)	66.8, CH	67.1, CH	4.49, q (6.4)	66.9, CH

6"	17.1, CH_3	1.14, d (6.4)	17.0, CH_3	17.2, CH_3	1.09, m	17.0, CH_3
1"	99.6, CH	4.8, d (3.1)	99.4, CH	100.4, CH	4.83, brs	100.2, CH
2"'	23.8, CH_2	1.5-2.2, m	23.6, CH_2	24.1, CH_2	1.5-2.2, m	23.9, CH_2
3"'	26.1, CH_2	1.5-2.2, m	26.0, CH_2	25.6, CH_2	1.5-2.2, m	25.5, CH_2
4"'	67.0, CH	3.65, brs	66.9, CH	67.4, CH	3.66, brs	67.5, CH
5"'	67.7, CH	4.39, q (6.3)	67.5, CH	68.1, CH	3.97, q (5.6)	68.0, CH
6"'	17.3, CH_3	1.06, d (6.3)	17.1, CH_3	17.2, CH_3	1.14, m	17.0, CH_3

Supplementary Table 3. ^{1}H (700 MHz) and ^{13}C NMR (175 MHz) spectral data of 8 and 9 in $CDCl_3$.

	8		9		epelymycin D (ε-rhodomycin)[2]
	δ_C	δ_H	δ_C	δ_H	δ_C
1	119.7, CH	7.83, d (7.3)	119.9, CH	7.88, d (6.9)	119.6, CH
2	137.1, CH	7.68, d (7.9)	137.3, CH	7.72, t (7.8)	137.0, CH
3	124.8, CH	7.28, d (8.4)	125.1, CH	7.29, d (7.7)	124.8, CH
4	162.6, C		162.8, C		162.7, C
4a	116.2, C		116.2, C		116.1, C
5	190.6, C		191.0, C		190.8, C
5a	111.2, C		111.8, C		111.6, C
6	157.1, C		156.9, C		156.9, C
6a	135.0, C		135.8, C		136.0, C
7	70.9, CH	5.19, brs	71.5, CH	5.25, d (2.5)	71.1, CH
8	37.1, CH_2	2.02, m; 1.80, m	33.5, CH_2	2.35, d (15.0); 2.24, dd (15.0, 4.2)	33.3, CH_2
9	69.6, C		71.5, C		71.3, C
10	36.6 CH_2	3.23, d (19.0); 2.53, d (19.0)	52.2, CH	4.29, s	52.2, CH
10a	138.6, C		135.5, C		135.5, C
11	157.3, C		157.0, C		156.9, C
11a	110.3, C		111.5, C		111.3, C
12	186.2, C		186.4, C		186.2, C
12a	133.5, C		133.5, C		133.4, C
13	35.4, CH_2	1.77, m; 1.68, m	32.4, CH_2	1.84, dq (14.1, 7.3); 1.45, dq (14.1, 7.3)	32.4, CH_2
14	7.5, CH_3	1.08, t (7.4)	6.9, CH_3	1.12, t (7.3)	6.8, CH_3
15	-	-	171.4, C		171.3, C
16	-	-	52.6, CH_3	3.71, s	52.4, CH_3
1'	100.8, CH	5.53, s	101.1, CH	5.57, d (3.4)	101.5, CH
2'	27.8, CH_2	2.03, m; 1.92, m	27.3, CH_2	2.14, m; 1.89, dd (12.8, 3.8)	29.4, CH_2
3'	61.1, CH	2.58, brs	61.5, CH	2.67, m	59.4, CH
4'	65.9, CH	3.84, brs	65.8, CH	3.92, br s	66.0, CH
5'	67.1, CH	4.14, q (6.4)	67.4, CH	4.09, q (6.3)	66.6, CH
6'	17.0, CH_3	1.37, d (6.3)	16.9, CH_3	1.37, d (6.4)	17.0, CH_3
3'-$N(CH_3)_2$	41.9, CH_3	2.41, s	41.9, CH_3	2.50, s	42.0, CH_3

Supplementary Table 4. Bacteria and plasmids used in this study.

Strains	Description	Reference or source
E. coli		
DH5α	Host strain for general clone	Stratagene
ET12567	*dam, dcm, hsdS, cat, tet*	3
BW25113	K12 derivative: *araBAD, rhaBAD*	3
BL21(DE3)	F-*ompT hsdS gal dcm* (DE3)	Novagen
**Plasmids**		
pET28a(+)	Kan^R, expression vector	Novagen
pIJ790	Cml^R, including λ-RED (*gam, bet, exo*) for PCR-targeting	3
pIJ773	*aac(3)IV* (Apr^R), *oriT*	3
pUZ8002	*tra, neo, RP4*	3
p11D5	SCSIO 1666 genomic library cosmid	This study
pJuCytA	p11D5 cosmid derivative where *cytA* was disrupted by *aac(3)IV-oriT* fragment using primers cytA-delF and cytA-delR	This study
**Mutant strain**		
Δ*cytA*	*cytA* gene disrupted mutant of SCSIO 1666	This study

Supplementary Table 5. Primer pairs used for mutant producer strain construction, verification, and protein overexpression.

Primers	Sequence (5′-3′)
cytA-delF	GCCACGCATGTGCGCCGCTACGTGGCCAGCGAGGGCGCGATTCCG GGGATCCGTCGACC
cytA-delR	CACGACGACGGGGATCTTCCGGTCGGTCTTCTTCTGGTATGTAGGC TGGAGCTGCTTC
cytA-testF	GACAGCCCGACCGGATGGGTC
cytA-testR	TGCGGGCCCCTGTGGCTCCA
cytA-expF	GGAATTCCATATGGACGAACAGGACGAGGTGCT
cytA-expR	CCGGAATTCTCATCCCCGTGCGGGCCCCT

Supplementary References

1. Johdo, O. *et al*. Anthracycline metabolites from *Streptomyces violaceus* A262. I. Isolation of antibiotic-blocked mutants from *Streptomyces violaceus* A262. *J. Antibiot.* **44**, 1110-1120 (**1991**).
2. Johdo, O. *et al*. Anthracycline metabolites from *Streptomyces violaceus* A262. II. New anthracycline epelmycins produced by a blocked mutant strain SU2-730. *J. Antibiot.* **44**, 1121-1129 (**1991**).
3. Gust, B.; Kieser, T. & Chater, K. F. REDIRECT technology: PCR-targeting system in *Streptomyces coelicolor*. The John Innes Centre, Norwich, United Kingdom (**2002**).
