# Supplementary material for: CytA, a reductase in the cytorhodin biosynthesis pathway, inactivates anthracycline drugs in Streptomyces
Source: Commun Biol. 2019 Dec 6;2:454. doi: 10.1038/s42003-019-0699-5 (PMC6897945; doi:10.1038/s42003-019-0699-5)
Supplement: Supplementary file 2 — Description of Additional Supplementary Files [file 42003_2019_699_MOESM2_ESM.docx]

Description of Additional Supplementary Items

Supplementary Data 1. Source Data for Table 1 and Supplementary figure 6
